# Molecular Design of a Naturally Derived Hemostatic Sealant with Prolonged Antimicrobial Activity for Repairing Elastic Organ Injuries

**DOI:** 10.1002/advs.202506466

**Published:** 2025-09-14

**Authors:** Saumya Jain, Avijit Baidya, Joshua A. Boys, George Z. Cheng, Taichiro Imahori, Naoki Kaneko, Nasim Annabi

**Affiliations:** ^1^ Department of Chemical and Biomolecular Engineering University of California Los Angeles Los Angeles CA 90095 USA; ^2^ Division of Cardiothoracic Surgery Department of Surgery University of California San Diego La Jolla CA 92093 USA; ^3^ Division of Pulmonary Critical Care and Sleep Medicine Department of Medicine University of California San Diego La Jolla CA 92093 USA; ^4^ Division of Radiological Sciences David Geffen School of Medicine University of California Los Angeles Los Angeles CA 90095 USA; ^5^ Department of Bioengineering University of California Los Angeles Los Angeles CA 90095 USA

**Keywords:** antibacterial, bioadhesive, elastic, hemostatic, sealant

## Abstract

Hemorrhaging injuries on dynamic internal organs present significant clinical burdens due to their complex nature. To address therapeutic challenges, an injectable, photocrosslinkable, and multifunctional bioadhesive hydrogel comprising methacrylated gelatin (GelMAG), methacrylated dopamine (DMA), and poly(diallyldimethylammonium chloride) (pDDA), named GDP, is engineered. The hydrogel combined underwater adhesion, antimicrobial activity, and hemostatic performance with high elasticity, biomimetic stiffness, and biocompatibility. The GDP hydrogel displayed >200% elongation and ≈50 kPa Young's modulus in tensile tests. The bioadhesive strongly adhered (>40 kPa strength) to skin, outperforming commercial sealants Coseal and Evicel, and could seal various sizes and shapes of injuries created on explanted pig lungs. Broad‐spectrum and long‐term in vitro antibacterial activity is noted. During in vivo rat liver puncture and tail amputation, GDP achieved significantly reduced blood loss (≈65%) compared to commercial hemostat Surgicel in some cases. In a clinically relevant porcine lung laceration model, GDP sealed large defects and reduced blood loss by 45–55% compared to Surgicel and hemostatic sealant TISSEEL. It also supported enhanced wound closure and tissue regeneration with minimal inflammation. Ultimately, these findings showcased the potential of GDP to act as an elastic, antibacterial, and hemostatic sealant for the repair of multi‐dimensional traumatic injuries on soft, dynamic tissues.

## Graphical abstract

An injectable, photocrosslinkable hydrogel sealant—comprising GelMAG, DMA, and pDDA—demonstrated strong underwater adhesion, elasticity, biocompatibility, and broad‐spectrum antibacterial activity. This material rapidly sealed dynamic internal organ injuries and significantly reduced blood loss in vivo. Its superior performance over commercial products highlighted its potential for treating complex, multi‐dimensional injuries in soft and elastic tissues.

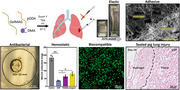

## 1 Introduction

Trauma is a major contributor to the global burden of disease, causing more than four million fatalities annually and imposing substantial economic strain.^[^ ^1^ ^]^ Survivors often suffer post‐traumatic complications, including coagulopathy, thromboembolism, infection, sepsis, organ failure, and stroke.^[^ ^2^ ^]^ Despite modern medical advances, treatment of trauma can easily be hampered by hemorrhage, infection, or inadequate wound closure. Current methods to induce hemostasis, such as gauze compression, infused dressings (e.g., chitosan, kaolin, zeolites), and transfusion of blood‐derived products, are beset by limitations like poor tissue adhesion, fiber shedding into the wound, secondary bleeding upon removal, risk of thrombosis or disease transmission, and reliance on cold storage or costly processing.^[^ ^3^, ^4^, ^5^, ^6^ ^]^

Polymeric biomaterials (sprays, sponges, foams) and hydrogels have emerged to address these shortcomings. While hydrogels can mimic native extracellular matrices and conform to injuries, they may exhibit suboptimal wet tissue adhesion (<20 kPa), insufficient elasticity, or require catalysts (UV light, oxidants, mechanical pressure) for activation, thereby compromising their clinical practicality for urgent use or on internal injuries.^[^ ^12^, ^13^, ^14^ ^]^ Efforts to enhance adhesion via catechol grafting on biopolymers, such as gelatin, have improved wet adhesion, but often at the expense of crosslinking efficiency and mechanical robustness.^[^ ^15^, ^16^ ^]^ Meanwhile, multifunctional systems incorporating silver nanoparticles, graphene oxide, or photothermal agents provide antibacterial function, but can suffer from metal ion toxicity, reactive oxygen species (ROS)‑related inflammatory responses, and requirements for instigating stimuli.^[^ ^17^, ^18^, ^19^ ^]^

Recent literature has explored multifunctional hydrogels for hemostasis and wound treatment, but they may have certain limitations that could hinder broad clinical translation. For example, a hydrogel based on methacrylated gelatin (GelMA) that was grafted with dopamine (DA) and mixed with quaternized chitosan and glycerol sponge achieved lap‐shear strengths of 125–138 kPa and hemostatic efficacy in rat tail and liver models; however, it relied on potentially immunogenic chitosan and lacked validation in large, pressurized organ models.^[^ ^7^ ^]^ In another case, a GelMA–DA photopolymerized patch was developed with impressive stretchability and adhesion (≈140% strain, 5700 J m^−^^3^ toughness), but it required alkaline‐activated dopamine oxidation and UV crosslinking, raising concerns over ROS and tissue safety.^[^ ^8^ ^]^ Furthermore, pre‐made patches may not be well‐suited for non‐compressible or irregularly shaped injuries. Similarly, multifunctional systems combining GelMA–DA with metal ions like Fe^3^⁺ showed efficiency in wound healing but depend on photothermal activation and posed risks of metal‐ion cytotoxicity.^[^ ^22^ ^]^ Other catechol‐based adhesives, such as GelMA combined with hyaluronic acid (HA) and DA (GDHA), polydopamine (PDA)–Fe^3^⁺ composites, and DA oligomer‐intercalated GelMA, achieved strong wet adhesion (>100 kPa) and biocompatibility, yet omitted hemostasis, antibacterial functionality, or in vivo testing in organs under physiological pressure.^[^ ^23^, ^24^ ^]^ More advanced injectable sealants combining GelMA with antibacterial metal oxides (e.g., zinc ferrite silicate composites) improved bacterial suppression and lung sealing *ex vivo*, but still relied on nanoparticle‐mediated ion release and photopolymerization.^[^ ^24^ ^]^ Our lab has previously reported a lung sealant based on methacryloyl‐modified tropoelastin (MeTro), but its broader application could be constrained by challenges related to the scalability and cost of the difficult‐to‐synthesize material.^[^ ^9^ ^]^ Therefore, a multifunctional hydrogel sealant that is strongly adhesive in wet environments, elastic, hemostatic, antibacterial, biodegradable, tissue‐compatible, and validated across both small and large animal models has not yet been realized.

We hypothesize that a multifunctional hydrogel engineered to simultaneously support hemostasis, tissue adhesion, and antimicrobial activity can address the complex demands for traumatic wound care. To achieve this, we developed a composite system comprising glycidyl methacrylated gelatin (GelMAG) that was grafted with methacrylic anhydride‐modified dopamine (DMA), which enabled covalent and noncovalent wet tissue adhesion. We also incorporated poly(diallyldimethylammonium chloride) (pDDA) that conferred contact‐activated antibacterial activity and additional hemostatic efficacy through its quaternary ammonium functionality, without the need for metal ions, ROS, UV/heat triggers, or blood products. The composite GelMAG, DMA, pDDA hydrogel, herein GDP, could be precisely tuned in formulation to match the viscoelasticity of soft tissue, and the biodegradable matrix degrades cleanly without eliciting inflammation.

We performed a thorough validation of GDP tissue adhesion ability, including in vitro pig skin adhesion and burst pressure testing, ex vivo sealing of injuries on ventilated pig lungs, and in vivo sealing evaluations using various animal models. Prolonged antibacterial efficacy was assessed against Gram‐negative *Pseudomonas aeruginosa* and methicillin‐resistant *Staphylococcus aureus* (MRSA) over seven days. Hemostasis was evaluated in vitro on human whole blood before in vivo tests using both rat liver puncture and rat tail amputation models. Biocompatibility was assessed in vitro on NIH 3T3 cells, and in vivo cytotoxicity and biodegradation studies were conducted using a rat subcutaneous implantation model. Uniquely, the multifunctional GDP was then evaluated in a two‐week pig lung laceration model, upon which it demonstrated air‐tight sealing, rapid hemostasis, cytocompatibility, and tissue regeneration.

To our knowledge, no existing adhesive hydrogel matches the fundamental material design, functional integration, and in vivo validation exhibited by GDP. Its performance across biological, mechanical, and translational domains firmly establishes the potential of GDP as a platform for trauma repair in both internal, pressurized organs and external elastic tissues.

## 2 Results

### 2.1 Formation and Physical Characterization

To address the critical requirements of hemostatic sealants for the repair of multi‐dimensional injuries, we designed a tough and elastic hydrogel, named GDP, that exhibited hemostatic and antimicrobial properties as well as strong wet tissue adhesion. As the backbone of the sealant, gelatin provided tissue mimicking elasticity and mechanical softness as well as biocompatibility. A dopamine derivate was also incorporated because of its role in the durable adhesion of marine mussels to underwater surfaces.^[^ ^4^ ^]^ Lastly, pDDA polyelectrolyte was introduced due to its ion‐saturated backbone that permits blood clotting and bacterial membrane disruption.^[^ ^5^ ^]^ The combination of all three inexpensive and easily scalable components produced a sealant with well‐balanced properties. For these reasons, the engineered sealant could be suitable for a broad range of injuries without concern for size, depth, or location.

**Figure 1:**
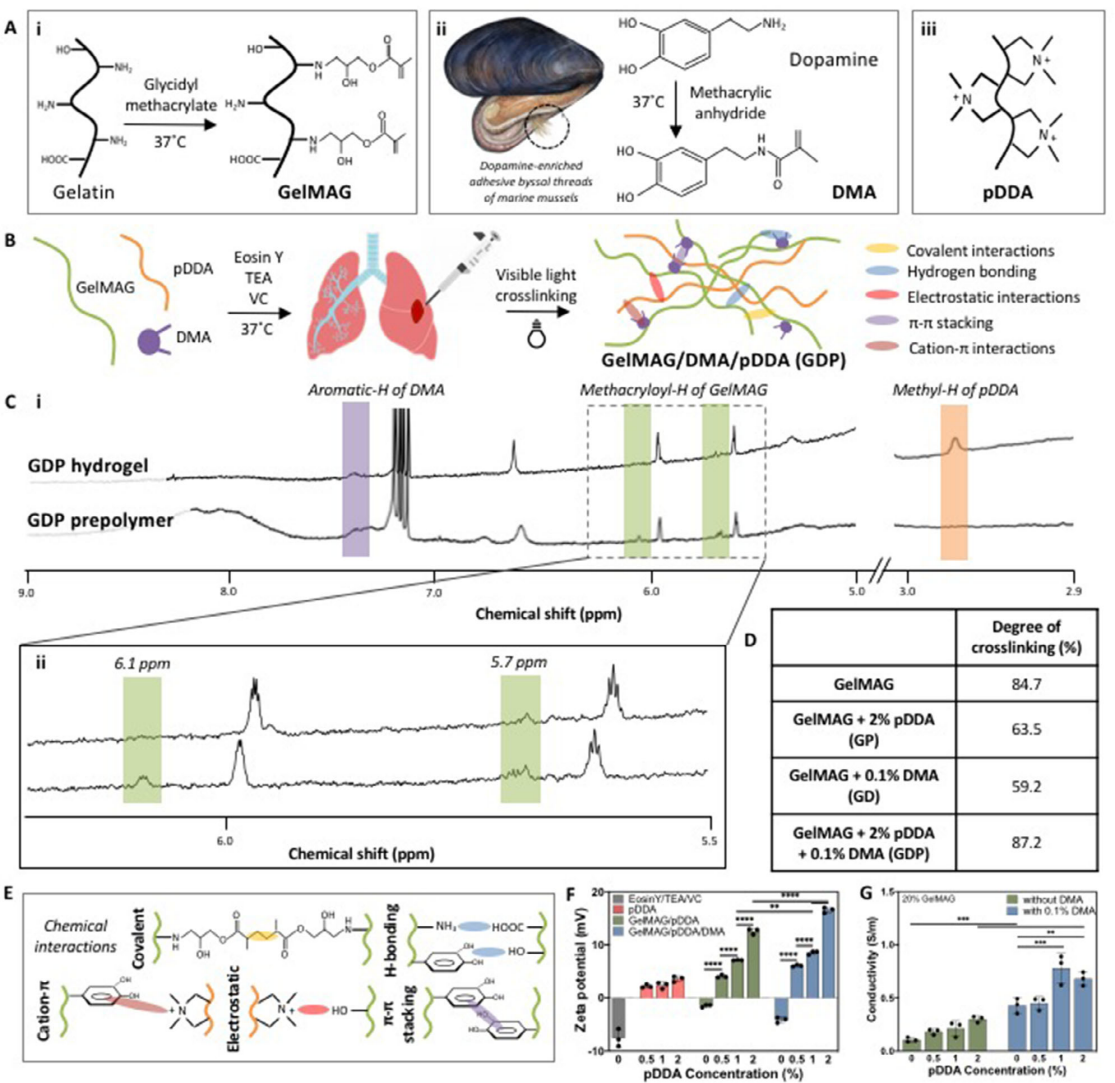
GDP hydrogel synthesis and characterization. A) Chemical structure of GelMAG synthesized through a one‐step conjugation reaction with glycidyl methacrylate and gelatin (left box), DMA synthesized by covalent attachment of methacrylic anhydride to dopamine (middle box), and pDDA (right box). B) Schematic of hydrogel preparation by physical mixing of GelMAG, DMA, and pDDA in Eosin Y/TEA/VC photoinitiator solution, application of thermosensitive prepolymer solution to an elastic organ injury, and in situ polymerization with visible light to form the GPD hydrogel. C) ^1^H NMR of (i) GDP prepolymer and hydrogel depicting aromatic protons of DMA (highlighted in purple), methyl protons of pDDA (highlighted in orange), and methacryloyl protons of GelMAG at 5.7 and 6.1 ppm (highlighted in green). D) Degree of crosslinking within hydrogels made of pure GelMAG, GelMAG with 2% (v/v) pDDA (GP), GelMAG with 0.1% (w/v) DMA (GD), and GelMAG with 0.1% (w/v) DMA and 2% (v/v) pDDA (GDP). E) Various chemical interactions within the crosslinked GDP composite. F) Zeta potential of GDP prepolymers with varying amounts of pDDA and DMA prepared in Eosin Y/TEA/VC photoinitiator solution. G) Conductivity of GDP hydrogels with varying amounts of pDDA and DMA. Data are represented as mean ± SD. Analysis by two‐way ANOVA with Tukey's post‐hoc multiple comparisons test. ^**^ *P* < 0.01, ^***^ *P* < 0.001, ^****^ *P* < 0.0001. n=3 biological replicates per group.

The GDP hydrogel was formed by first synthesizing its constituents: GelMAG and DMA. GelMAG was synthesized through a one‐step reaction between gelatin and glycidyl methacrylate^[^ ^10^ ^]^ (**Figure** **1**A). The synthesis of GelMAG was confirmed by proton nuclear magnetic resonance (^1^H NMR) spectroscopy through the emergence of peaks at 5.74 and 6.13 ppm corresponding to the two vinylic methacryloyl protons (Figure S1, Supporting Information). Furthermore, the amine protons on GelMAG lysine residues, presented as a peak at 2.78 ppm, indicated a degree of methacryloyl substitution of ≈50%. Similarly, DMA was synthesized through a reaction between dopamine hydrochloride and methacrylic anhydride (MA) (Figure 1A). Methacryloyl proton peaks at 5.33 and 5.64 ppm confirmed the synthesis of DMA (Figure S2, Supporting Information). Lastly, we introduced pDDA to provide both antimicrobial and hemostatic properties to the resulting hydrogel (Figure 1A).

The prepolymer solution of GelMAG, DMA, and pDDA was prepared with photoinitiators Eosin Y, triethanolamine (TEA),^[^ ^6^ ^]^ and *N*‐vinylcaprolactum (VC), and the GDP hydrogel was formed after 4 min of photocrosslinking with visible light exposure (Figure 1B). The ^1^H NMR spectra of the GDP precursor containing 20% (w/v) GelMAG, 0.1% (w/v) DMA, and 2% (v/v) pDDA depicted the presence of methacryloyl protons on GelMAG at 5.70 and 6.08 ppm (shown in green), DMA aromatic protons at 7.44 ppm (shown in purple), and the pDDA methyl protons at 2.97 ppm (shown in orange) (Figure 1C). Upon photocrosslinking, the methacryloyl proton peaks of GelMAG largely diminished, indicating covalent bond formation between adjacent moieties and, thereby, hydrogel formation. However, the presence of either pDDA or DMA in GelMAG‐pDDA (GP) or GelMAG‐DMA (GD) hydrogels, respectively, seemed to reduce the extent of methacryloyl peak consumption, indicating lower degrees of crosslinking (Figure S3, Supporting Information). Accordingly, the GelMAG hydrogel was characterized to have an 85% degree of crosslinking, which was higher than the 64% or 59% degree of crosslinking in the GP or GD gels, respectively (Figure 1D). Nevertheless, when all three components were combined to form the GDP precursor, there was a higher (87%) degree of crosslinking in the resulting hydrogel.

Numerous physical and chemical interactions between the constituents, including covalent bonding between GelMAG methacryloyl residues as well as cation‐π interactions between pDDA and DMA, likely occurred during the crosslinking of the GDP sealant (Figure 1E). The possibility of physical crosslinking in the hydrogels via electrostatic interactions was assessed through zeta potential measurements of various prepolymer solutions (Figure 1F). The photoinitiator solution (EosinY/TEA/VC), GelMAG, and GD prepolymers all exhibited negative zeta potentials, while pure pDDA as well as prepolymer solutions containing pDDA had increasingly positive zeta potentials corresponding to higher pDDA concentration. Due to the high surface charge of pDDA‐containing solutions, there were likely electrostatic interactions in their resulting hydrogels. In addition to increasing the extent of physical crosslinking, the presence of both pDDA and DMA also impacted the ionic conductivity of the GDP composite. Compared to GelMAG (0.11 ± 0.017 S m^−1^), GD hydrogels exhibited 0.43 ± 0.065 S m^−1^ conductivity, likely due to the ability of anionic DMA to form hydrogen bonding and electrostatic interactions with water (P < 0.001) (Figure 1G). After the addition of 1% and 2% (v/v) pDDA to the GDP hydrogels, their conductivities increased to 0.78 ± 0.13 S m^−1^ (P < 0.001) and 0.68 ± 0.051 S m^−1^ (P < 0.01), respectively. In addition to their higher zeta potential compared to gels without pDDA, the GDP hydrogels likely exhibited higher ionic conductivity due to the doping effect that DMA had on pDDA.^[^ ^11^ ^]^ Since the conductivity of GDP containing 2% (v/v) pDDA was within the range of native electroactive tissues like muscle (0.04–0.5 S m^−1^), cardiac (0.5 S m^−1^), and nerve (0.08–1.3 S m^−1^), it could ultimately facilitate cellular communication and tissue regeneration in their diverse biophysical environments.^[^ ^12^ ^]^ In order to validate the potential chemical interactions that could occur within the hydrogel upon photocrosslinking, we conducted Fourier transform infrared (FTIR) spectroscopy on GelMAG, GP, GD, and GDP gels (Figure S4, Supporting Information). There were consistent peaks across all samples, indicating that modifying GelMAG with DMA or pDDA did not alter the bulk chemical backbone. In particular, all hydrogels contained the broad peak ≈3300 cm^−1^, representative of O‐H or N‐H hydrogen bond stretching vibrations. Also, all samples contained the characteristic amide peaks (1650 and 1540 cm^−1^) that are associated with gelatin. GDP seemed to have lower transmittance of a peak at 600 cm^−1^, which is absent in all other formulations and may be due to non‐covalent bonding between the aromatic group of DMA and the ammonium group of pDDA. The bands corresponding to the resulting cation‐π bonds could exist in the fingerprint region.^[^ ^13^ ^]^

### 2.2 Mechanical Characterization

**Figure 2:**
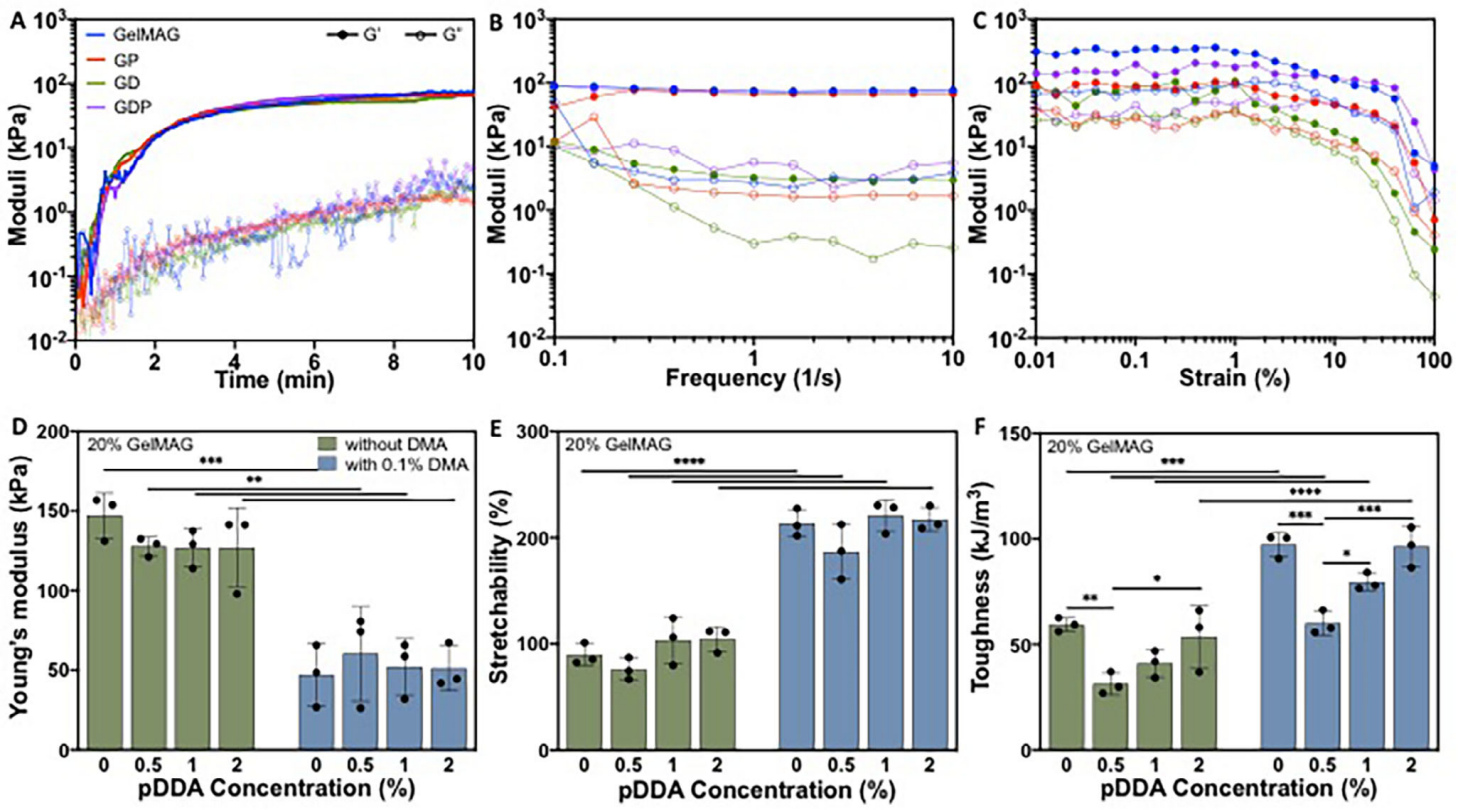
Rheological and mechanical characterization of GDP sealant. A) Time sweep of precursor solutions of GelMAG, GP (with 2% v/v pDDA), GD (with 0.1% w/v DMA), and GDP (with 0.1% w/v DMA and 2% v/v pDDA) that were crosslinked with visible light for 4 min, depicting storage modulus (G′) and loss modulus (G″) over 10 min. B) Frequency sweep displaying the elastic behavior of engineered hydrogels. C) Strain sweep measurements depicting the linear viscoelastic region of all hydrogel formulations. D) Tensile Young's modulus of GDP hydrogels prepared with varying concentrations of pDDA and DMA. E) Hydrogel stretchability in response to varying concentrations of pDDA and DMA. F) Toughness of all hydrogel formulations.

Hydrogel sealants developed for sealing soft tissue injuries must adapt to terrains of dynamic rigidity and mobility in order to prevent mechanical mismatch and dehiscence, as well as to promote tissue repair and regeneration.^[^ ^14^ ^]^ Biomimetic mechanical properties that are suitable for soft and elastic organs like the lungs fall in the range of 1–5 kPa Young's modulus^[^ ^15^ ^]^ and up to 128% extensibility, which many engineered sealants may not be able to accommodate. Therefore, to assess the conformability of GDP sealants to injuries on dynamic organs, their mechanical properties were characterized using a rheometer and an Instron mechanical tester. Time sweet rheological measurements were first conducted to monitor the evolution of the storage modulus (G′) and loss modulus (G″) during hydrogel formation (**Figure** **2**A). After 4 min or crosslinking with visible light, the moduli of all hydrogel formulations stabilized at ≈50–70 kPa, indicating rapid and efficient crosslinking. Since no visible difference was noted between GelMAG, GP (with 2% v/v pDDA), GD (with 0.1% w/v DMA), and GDP (with 0.1% w/v DMA and 2% v/v pDDA) hydrogels, it could be inferred that neither the bulk hydrogel mechanics nor the gelation kinetics were impacted by the additional components. On the other hand, the frequency sweep measurements revealed more substantial differences in mechanical behavior between the hydrogels (Figure 2B). While all samples exhibited higher G′ than G″ across a broad frequency range, indicating predominantly elastic behavior, the GelMAG, GP, and GDP hydrogels maintained the highest and most frequency‐independent G′ values, emphasizing their structural integrity throughout dynamic stress and reduced susceptibility to network relaxation. Similarly, strain sweep measurements provided insight into the structural resilience of the hydrogels. While GelMAG, GP, and GDP hydrogels maintained their elastic moduli within the linear viscoelastic region until 40–50% strain before structure breakdown, the GD hydrogel underwent mechanical failure ≈15% strain (Figure 2C). Extended linear viscoelastic regions could indicate higher crosslinking density and network cohesion, allowing the materials to accommodate substantial deformation before failure. Their ability to withstand higher strain supports their application in surgical settings where they must endure dynamic tissue deformations.

Tensile testing was conducted to further examine the response of the engineered hydrogels to biologically relevant mechanical stimuli. A pure 20% (w/v) GelMAG hydrogel possessed a Young's modulus of 147 ± 13 kPa, whereas GD and GDP hydrogels exhibited 47 ± 18 kPa (P < 0.001) and 51 ± 12 kPa (P < 0.01) moduli, respectively (Figure 2D). The reduced Young's moduli brought the engineered hydrogels closer to the range of native soft tissues (e.g., breast, muscle, liver).^[^ ^16^ ^]^ The mechanical softness of the GDP sealant was directly proportional to its extensibility. While pure GelMAG exhibited 90 ± 8.4% stretchability, the GDP hydrogel containing 2% (v/v) pDDA withstood 217 ± 8.4% stretching until failure, making it an optimal candidate for lung injury sealing (P < 0.0001) (Figure 2E). The addition of pDDA and DMA also bolstered the toughness of the engineered hydrogels. The 59 ± 2.1 kJ m^−3^ toughness of pure GelMAG increased to 97 ± 5.3 kJ m^−3^ for the GD hydrogel (P < 0.001) (Figure 2F). After adding 0.5% (v/v) pDDA to GDP sealant, the toughness dropped to 60 ± 5.2 kJ m^−3^ (P < 0.001), and after increasing pDDA concentration in GDP to 2% (v/v), the toughness rose to 96 ± 8.3 kJ m^−3^ (P < 0.001). Although adding DMA resulted in higher toughness, the ultimate strength of GD (102 ± 4.68) was lower than that of pure GelMAG (140 ± 13 kPa) (P < 0.01) (Figure S5, Supporting Information). Adding 0.5% (v/v) pDDA to GD hydrogel further reduced the ultimate strength, but increasing pDDA concentration to 2% (v/v) restored its properties so that the GDP sealant retained 101 ± 6.5 kPa ultimate strength. Furthermore, since the lungs undergo continuous expansion and contraction, we assessed the extent of energy loss of the GDP hydrogels after cyclic deformation. Compared to the 10 ± 0.48% energy loss of pure GelMAG after 12 cycles of compression, the GDP sealant exhibited 22 ± 1.3% energy loss, both of which are relatively low (P < 0.05) (Figure S6, Supporting Information).

### 2.3 In Vitro and Ex Vivo Adhesion Properties

**Figure 3:**
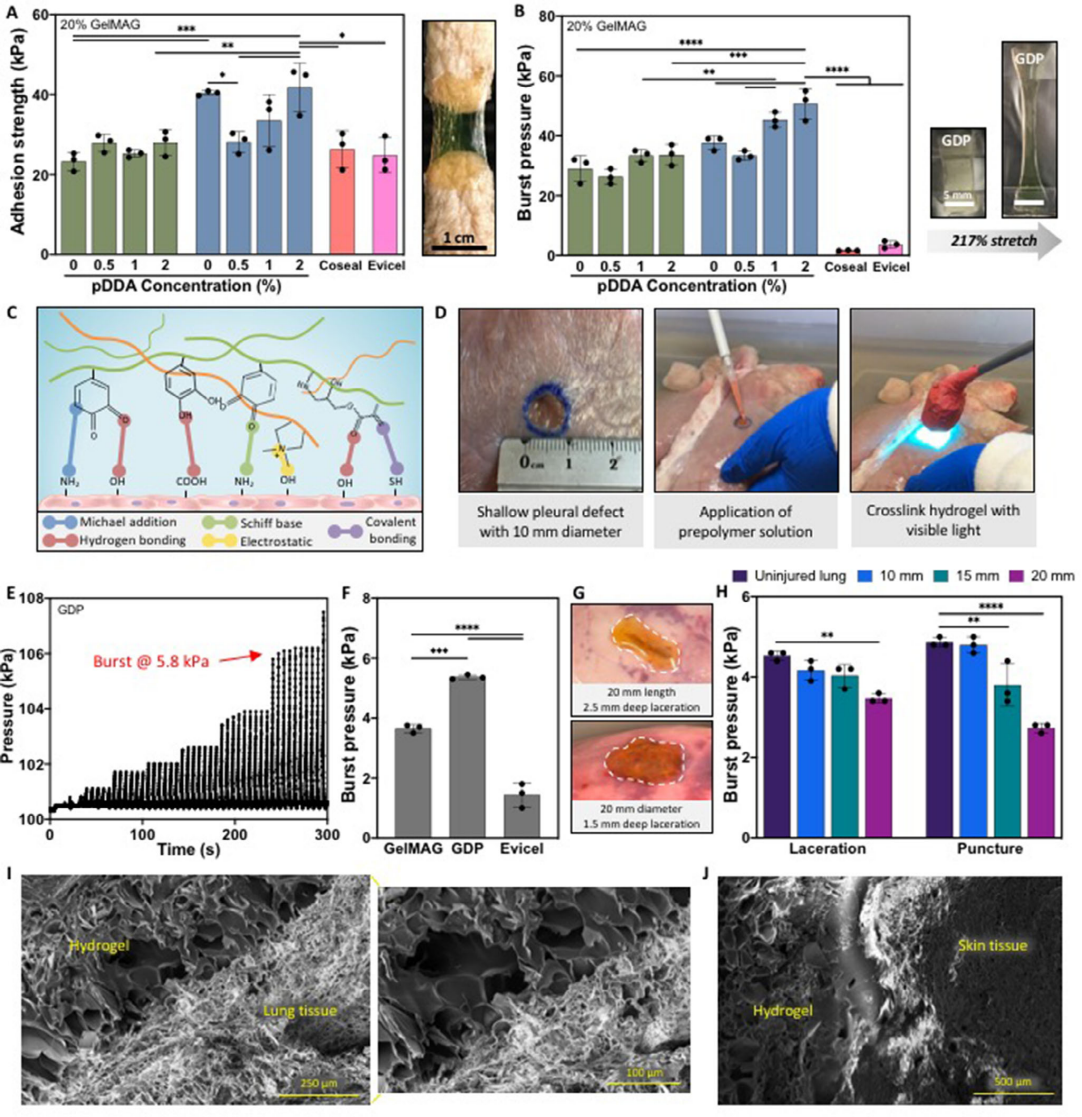
In vitro and ex vivo adhesion characterization of GDP sealant. A) In vitro adhesion strength based on the wound closure test on porcine skin and B) in vitro burst pressure on punctured collagen sheet using GDP hydrogels with varying amounts of pDDA and DMA, as well as commercial controls, Coseal and Evicel.^[^ ^17^ ^]^ C) Schematic of potential chemical interactions occurring between the GDP hydrogel and tissue surface. D) Setup for an *ex vivo* lung injury model involving attaching an unpunctured pig lung to a ventilator and creating a 10 mm wide pleural defect, applying prepolymer solution of GelMAG or GDP, photocrosslinking with visible light to form hydrogel sealants, and E) ventilating the lung with gradually increasing pressures until the hydrogel bursts. F) Ex vivo burst pressures of hydrogels and Evicel as a commercial control. Analysis by one‐way ANOVA with Tukey's post‐hoc multiple comparisons test. G) Representative images of large lacerations (20 mm length and 2.5 mm depth) or large punctures (20 mm diameter and 2.5 mm depth) created on explanted pig lungs and sealed with GDP hydrogel. H) Ex vivo burst pressure of GDP hydrogel after sealing various sizes of lacerations and punctures compared to the unpunctured lung. Representative SEM images from the cross‐section of GDP sealant adhered onto porcine I) lung and J) skin tissues. Data are represented as mean ± SD. Analysis by two‐way ANOVA with Tukey's post‐hoc multiple comparisons test. ^*^ *p* < 0.05, ^**^ *p* < 0.01, ^***^ *p* < 0.001, ^****^ *p* < 0.0001. n = 3 biological replicates per group.

Alongside mechanical conformability, robust adhesion of hydrogels to wet biological surfaces is essential for reliable wound sealing. To assess the adhesive properties of the GDP sealant, standard in vitro wound closure (ASTM F2458) and burst pressure (ASTM F2054) tests were performed.^[^ ^17^, ^18^ ^]^ While pure GelMAG possessed an adhesion strength of 23 ± 1.9 kPa on pig skin, the catechol‐containing GD sealant exhibited a higher adhesion strength at 40 ± 0.04 kPa (P < 0.001) (**Figure** **3**A). After the introduction of pDDA to the GDP hydrogels, the adhesion strength decreased to 28 ± 2.2 kPa for GDP with 0.5% (v/v) pDDA (P < 0.05) but increased to 42 ± 5.7 kPa for GDP with 2% (v/v) pDDA (P < 0.01). The highly extensible and adhesive GDP sealant containing 2% (v/v) pDDA exhibited higher adhesion strength on porcine skin tissue compared to both Coseal (26 ± 4.3 kPa) and Evicel (25 ± 3.8) (P < 0.05). Adhesion energy was also found to be higher for GDP hydrogels (18 ± 3.6 J m^−2^) compared to GelMAG (7.6 ± 0.11 J m^−2^) or various GP samples (P < 0.05) (Figure S7, Supporting Information). Similar trends were observed during the burst pressure test using punctured and pressurized collagen sheets. When the sheet was sealed with pure GelMAG, it resulted in a burst pressure of 29 ± 3.6 kPa, while treatment with GD and GDP (with 2% v/v pDDA) sealants caused burst pressures of 38 ± 2.0 kPa and 51 ± 4.6 kPa (P < 0.0001), respectively (Figure 3B). The GDP sealant reported significantly higher burst pressure compared to Coseal (1.7 ± 0.11 kPa) and Evicel (3.7 ± 0.96) (P < 0.0001). While GelMAG can form covalent and hydrogen bonding interactions with tissue moieties (e.g., hydroxyl, amino, thiol groups), there is more potential for bioadhesive/tissue interactions (e.g., Michael addition, Schiff base, electrostatic interactions) with the presence of DMA and pDDA (Figure 3C).

The adhesive properties of the GDP sealants were further characterized using ex vivo burst pressure tests on freshly isolated pig lungs. The unpunctured lungs were attached to a ventilator, where a defect was made and sealed with the engineered hydrogels (Figure 3D). Then, the lungs were cyclically pressurized with air, and the hydrogel burst pressure was recorded using a Pasco Capstone software (Figure 3E). The optimized GDP sealant containing 0.1% (w/v) DMA and 2% (v/v) pDDA sealed a shallow pleural defect with 0.5 mm depth and 10 mm diameter with a burst pressure of 5.4 ± 0.06 kPa, which was significantly higher than the burst pressure of lungs sealed with GelMAG at 3.7 ± 0.12 kPa (P < 0.001) or Evicel at 1.4 ± 0.36 kPa (P < 0.0001) (Figure 3F). Larger and deeper defects mimicking clinically‐relevant trauma, such as stab or bullet wounds, were also created on explanted pig lungs in order to exemplify the efficacy of GDP sealant for sealing multi‐dimensional injuries. Lacerations of 2.5 mm depth and either 10, 15, or 20 mm length were prepared on the caudal (diaphragmatic) lobe and sealed with the optimal GDP sealant (Figure 3G). Punctures of 1.5 mm depth and either 10, 15, or 20 mm diameter were also created and sealed with GDP (Figure 3G). Since different lungs were used for each type of injury, the maximum pressure that each uninjured lung could withstand upon ventilation was measured using Pasco Capstone software. The GDP sealant effectively closed lacerations of 10 and 15 mm lengths with burst pressures of 4.2 ± 0.24 kPa and 4.0 ± 0.31 kPa, respectively, both of which were comparable to the normal maximum pressure of the lungs (4.5 ± 0.11 kPa) (Figure 3H). While sealing a 20 mm laceration, the GDP hydrogel exhibited a slightly lower burst pressure of 3.5 ± 0.10 kPa after multiple cycles of ventilation (Movie S1, Supporting Information). When the engineered sealant was applied to deep punctures, it sealed an injury of 10 mm diameter with 4.8 ± 0.18 kPa burst pressure, which was comparable to the maximum pressure of the uninjured lung (4.9 ± 0.11 kPa) (Figure 3H). The GDP sealant exhibited a slightly lower burst pressure on 15 mm diameter punctures and significant pressure drop on 20 mm punctures, but even the largest surface area wound could be sealed for several cycles before hydrogel detachment (Movie S2, Supporting Information).

In order to observe the robust adhesiveness of GDP on various biological surfaces, we conducted scanning electron microscopy (SEM)^[^ ^18^ ^]^ imaging on the intersections of GDP‐sealed tissues. For example, we crosslinked GDP on either freshly isolated soft porcine lung tissue (Figure 3I) or stiff porcine skin tissue (Figure 3J), and in either case, the engineered hydrogel displayed bioadhesion through mechanical interlocking with the tissue surfaces. The porous GDP matrix seamlessly integrated with the tissue, supporting its application as a surgical sealant for various topologies, regardless of their stiffness.

### 2.4 In Vitro Antibacterial Activity

**Figure 4:**
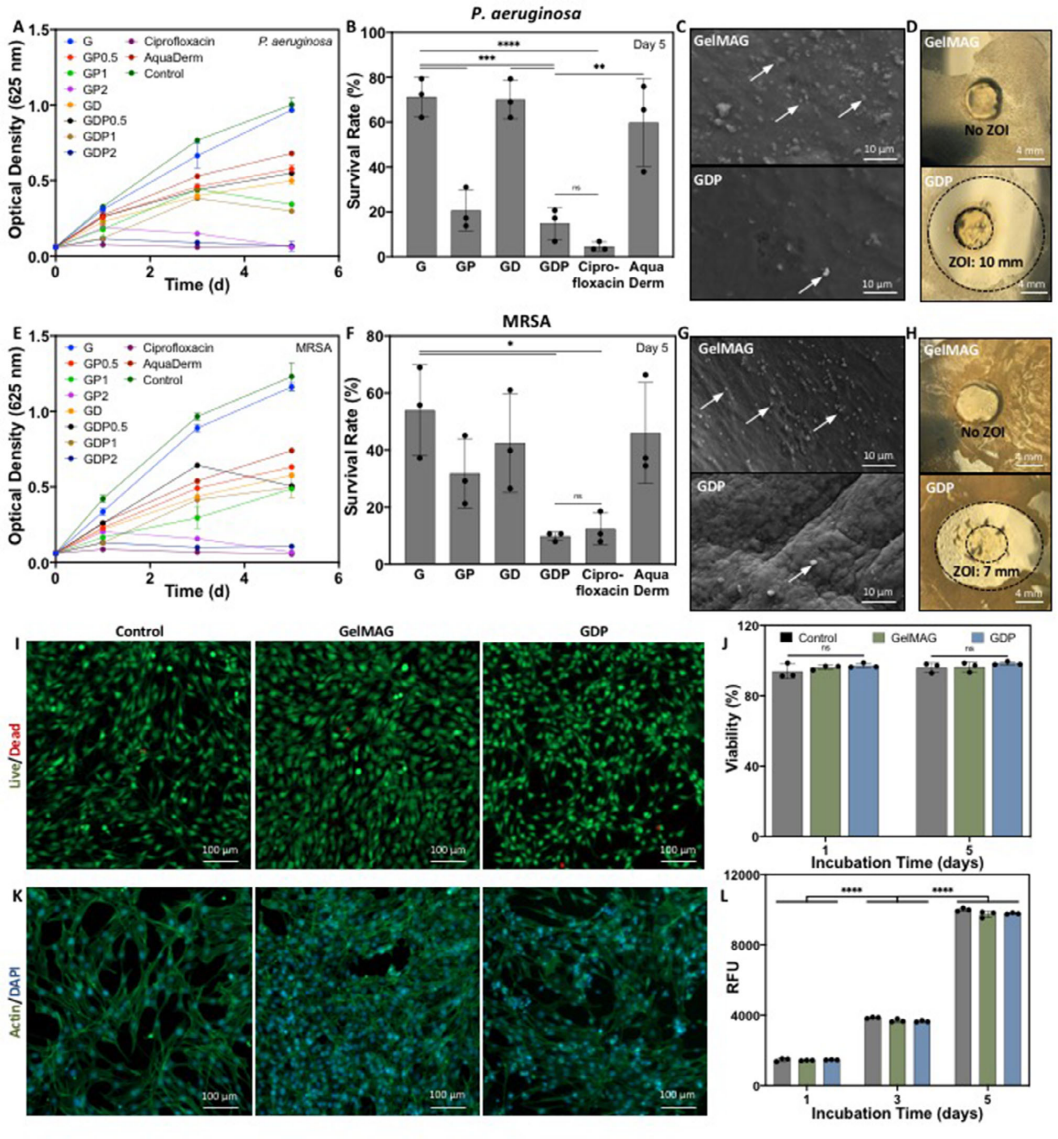
In vitro antibacterial properties and biocompatibility of GDP sealant. In vitro antimicrobial tests: A) Bacterial concentration measured by optical density (OD) at 625 nm of *P. aeruginosa* during 5 days of culture with either GDP hydrogels prepared with various concentrations of DMA and pDDA, broad‐spectrum antibiotic ciprofloxacin, or commercial wound dressing AquaDerm. B) Survival rate of *P. aeruginosa* after 5 days of treatment. C) Representative SEM images of GelMAG and GDP surfaces after 5 days of bacterial inoculation. D) Zone of inhibition (ZOI) formed by GelMAG or GDP against *P. aeruginosa*. E) OD measurements of MRSA during 5 days of culture with either GDP hydrogels prepared with various concentrations of DMA and pDDA, broad‐spectrum antibiotic ciprofloxacin, or commercial wound dressing AquaDerm. F) Survival rate of MRSA after 5 days of treatment. G) Representative SEM images of GelMAG and GDP surfaces after 5 days of bacterial inoculation. H) ZOI formed by GelMAG or GDP against MRSA. Negative control represented bacteria without hydrogel treatments. In vitro biocompatibility assessment: I) Representative live/dead stained images from NIH3T3 cells exposed to GelMAG or GDP through Transwell^®^ inserts for 5 days. Control cells were cultured without hydrogel exposure. J) Cellular viability after 1 and 5 days of incubation with either GelMAG or GDP hydrogels. K) Representative actin/DAPI stained images of NIH3T3 cells cultured with GelMAG or GDP for 5 days. L) Relative fluorescence unit (RFU) of cells cultured with GelMAG or GDP for 1, 3, and 5 days, assessed through PrestoBlue assay. Data are represented as mean ± SD. Analysis by two‐way ANOVA with Tukey's post‐hoc multiple comparisons test. ^*^ *p* < 0.05, ^**^ *p* < 0.01, ^****^ *p* < 0.0001. n = 3 biological replicates per group.

Traumatic injuries incur a significant risk of sepsis, which is known to increase medical costs three‐fold and is responsible for 10% of mortality after trauma.^[^ ^19^, ^20^ ^]^ To study the bactericidal nature of the GDP sealant, in vitro antibacterial tests were conducted against widely pathogenic *P. aeruginosa* and MRSA. GDP hydrogels prepared with varying concentrations of DMA (0–0.1% w/v) and pDDA (0–2% v/v) were incubated with either strain of bacteria for a period of 5 days, after which bacterial survival was measured either through spectrophotometric analysis of optical density (OD) at 625 nm or using the spread plate method to count colony forming units (CFU). The broad‐spectrum antibiotic ciprofloxacin was used against both bacteria as a positive control, and a commercial wound dressing, AquaDerm, was also compared to the engineered GDP hydrogels. While the untreated *P. aeruginosa* continued to proliferate throughout the incubation period, bacteria cultured with any formulation of GDP hydrogels, antibiotics, or AquaDerm experienced lower OD (**Figure** **4**A). Nevertheless, GelMAG, GP, GD, and GDP hydrogels containing lower concentrations of pDDA (0.5% v/v) could not inhibit bacterial growth, resulting in high OD (0.5–1.1) by day 5. AquaDerm was also unable to inhibit bacterial proliferation, causing ≈0.70 OD. On the other hand, GP and GDP hydrogels with higher concentrations of pDDA (1 or 2% v/v) exhibited low OD (0.1–0.2) at day 1, which further decreased by day 5 of the assay. However, ciprofloxacin seemed to completely diminish OD from the start of the incubation, indicating minimal bacterial growth. After the 5‐day culture, a spread plate method was utilized to count CFU (Figure S8A, Supporting Information), which was then used to calculate concentration (CFU/mL), survival rate, and log reduction of viable bacteria. The untreated *P. aeruginosa* control was 5.6 x 10^6^ CFU mL^−1^, whereas GDP treatment resulted in 8.7 x 10^5^ CFU mL^−1^ (P < 0.0001) (Figure S8B, Supporting Information), corresponding to a bacterial survival rate of 14.9 ± 6.4% (Figure 4B) and a log reduction of 0.98 ± 0.25 (Figure S8C, Supporting Information). The antibacterial efficacy of GDP was only slightly lower than that of ciprofloxacin, which experienced 2.7 x 10^5^ CFU mL^−1^, corresponding to 4.6 ± 1.9% survival (P = 0.99) and 1.5 ± 0.2 log reduction (P < 0.05). Bacteria treated with GDP exhibited a significantly lower survival rate compared to GelMAG (71.3 ± 8.2%, P < 0.001), GD (70.1 ± 7.8%, P < 0.001), or AquaDerm (59.8 ± 19%, P < 0.01). Furthermore, representative SEM images from the surface of GelMAG and GDP sealants after the antibacterial assay revealed far less *P. aeruginosa* present on the pDDA‐containing hydrogel (Figure 4C). GDP also caused a 10 mm zone of inhibition (ZOI) against *P. aeruginosa*, further demonstrating its bactericidal properties (Figure 4D). Culturing the hydrogels with MRSA elicited a similar response. While GelMAG exhibited similar OD to the untreated control, GP and GDP with higher pDDA concentrations stopped proliferation after 1 day (Figure 4E). They acted in a comparable manner to ciprofloxacin, which could immediately obstruct growth, and better than GelMAG, GD, GDP with lower pDDA concentration and AquaDerm treatments. After the 5‐day culture, the untreated MRSA control was at a concentration of 7.5 x 10^6^ CFU/mL, whereas GDP treatment led to 7.3 x 10^5^ CFU mL^−1^ (P < 0.0001) (Figure S8B, Supporting Information), corresponding to 9.7 ± 1.1% survival (Figure 4F) and 1.0 ± 0.07 log reduction (Figure S8C, Supporting Information). The survival rate of GDP‐treated MRSA was comparable to that of ciprofloxacin‐treated samples (12.4 ± 4.9%) (P = 0.99) and less than that of GelMAG (53.9 ± 15.7%) (P < 0.05). Similarly, SEM images revealed that GDP had less MRSA present on its surface compared to GelMAG (Figure 4G). In addition, GDP caused a 7 mm ZOI against MRSA (Figure 4H).

Since GDP and ciprofloxacin exhibited the strongest antibacterial properties, we cultured *P. aeruginosa* or MRSA with a range of concentrations of either antimicrobial agent to calculate the minimum inhibitory concentration (MIC) required to prevent bacterial growth. After a 24 h incubation, we determined that 1.6–2.3 µg mL^−1^ ciprofloxacin was sufficient for inhibiting either strain of bacteria (Figure S9, Supporting Information). On the other hand, since the active ingredient in GDP is only a small portion of the bulk hydrogel, a MIC of 2083–2500 µg mL^−1^ was required. We further assessed GDP's contact‐activation mechanism of bacterial resistance through live/dead staining of bacterial cultures after 5 days of treatment with GelMAG, GD, GP, GDP, ciprofloxacin, AquaDerm, or an untreated control (Figure S10, Supporting Information). For *P. aeruginosa*, the control, GelMAG, and GD samples showed predominantly green signals, indicating high bacterial survival. Bacteria treated with AquaDerm also showed a high green signal. In contrast, the GP and GDP‐treated bacteria displayed more pronounced red signals, comparable to those treated with ciprofloxacin, suggesting more prominent bactericidal activity. Likewise, for MRSA, the control, GelMAG, GD, and AquaDerm samples appeared to have a large number of live cells, whereas GP and GDP had a better antibacterial effect, similar to that of ciprofloxacin. Overall, live/dead staining revealed the potent antibacterial properties of GDP against both Gram‐negative and Gram‐positive bacteria. The strong antibacterial nature of the GDP sealant could be due to the presence of cationic pDDA, which could likely aggregate anionic bacterial cell membranes and disrupt their growth, as well as limit biofilm spreading.^[^ ^21^, ^22^ ^]^ As exemplified through zone of inhibition tests, pDDA likely leached out of the GDP matrix to interact with and inhibit bacterial growth. To assess the rate at which pDDA was released from the hydrogel, we measured the zeta potential of DI water that was incubated with the hydrogel over a period of time. The progressively increasing zeta potential confirmed that pDDA was likely released from the hydrogel during the initial water absorption process (Figure S11, Supporting Information). Solution containing GDP exhibited ≈1.3 mV zeta potential after 4 h of incubation and ≈10.5 mV after 48 h, both of which were significantly higher than the solution containing pure GelMAG (≈0.12 mV after 4 h, P < 0.05, and ≈3.2 mV after 48 h, p < 0.0001). Therefore, the GDP hydrogel could release pDDA for maximum bactericidal activity.

### 2.5 In Vitro Biocompatibility

Crucial limitations of most antibacterial and hemostatic sealants are their lack of biocompatibility^[^ ^8^, ^23^ ^]^ and biodegradability.^[^ ^24^ ^]^ For example, the aforementioned antibacterial hydrogels that could disrupt bacterial proliferation with the help of inorganic compounds could also likely cause cytotoxicity due to their generation of ROS. Herein, we assessed the in vitro biocompatibility of GDP sealant using a Transwell assay that exposed the hydrogels to NIH3T3 fibroblast cells through a membrane. The cells were cultured with either GelMAG or GDP sealants or without any hydrogels (negative control). Live/dead staining was conducted on day 1 (Figure S12, Supporting Information) and day 5 (Figure 4I) of culture, which depicted increasing amounts of live cells throughout the study. Cellular viability was quantified from live/dead stained images and revealed >95% viability for control cells as well as those cultured with GelMAG or GDP hydrogels (Figure 4J). Simultaneously, staining with F‐actinin and 4′,6‐diamidino‐2‐phenylindole dihydrochloride (DAPI) was conducted on day 1 (Figure S12, Supporting Information) and day 5 (Figure 4K) to assess cellular morphology via stained actin filaments, spreading behavior, and cell density through DAPI‐stained nuclei. As evidenced by the apparent increasing amounts of nuclei per unit area, the cell number after exposure to GDP increased from 408 ± 125 cells mm^−2^ at day 1 to 1998 ± 272 cells mm^−2^ at day 5 (p < 0.0001) (Figure S13, Supporting Information). The metabolic activity of the NIH3T3 cells exposed to engineered hydrogels was also assessed using a PrestoBlue assay, which depicted consistently increasing relative fluorescence units (RFU) for all tested groups (Figure 4L). Not only did the cells cultured with GDP sealant exhibit steady proliferation, but they also grew at a rate comparable to the control cells that were not incubated with the engineered hydrogels, thereby confirming the in vitro biocompatibility of GDP.

### 2.6 In Vitro and In Vivo Hemostatic Activity

**Figure 5:**
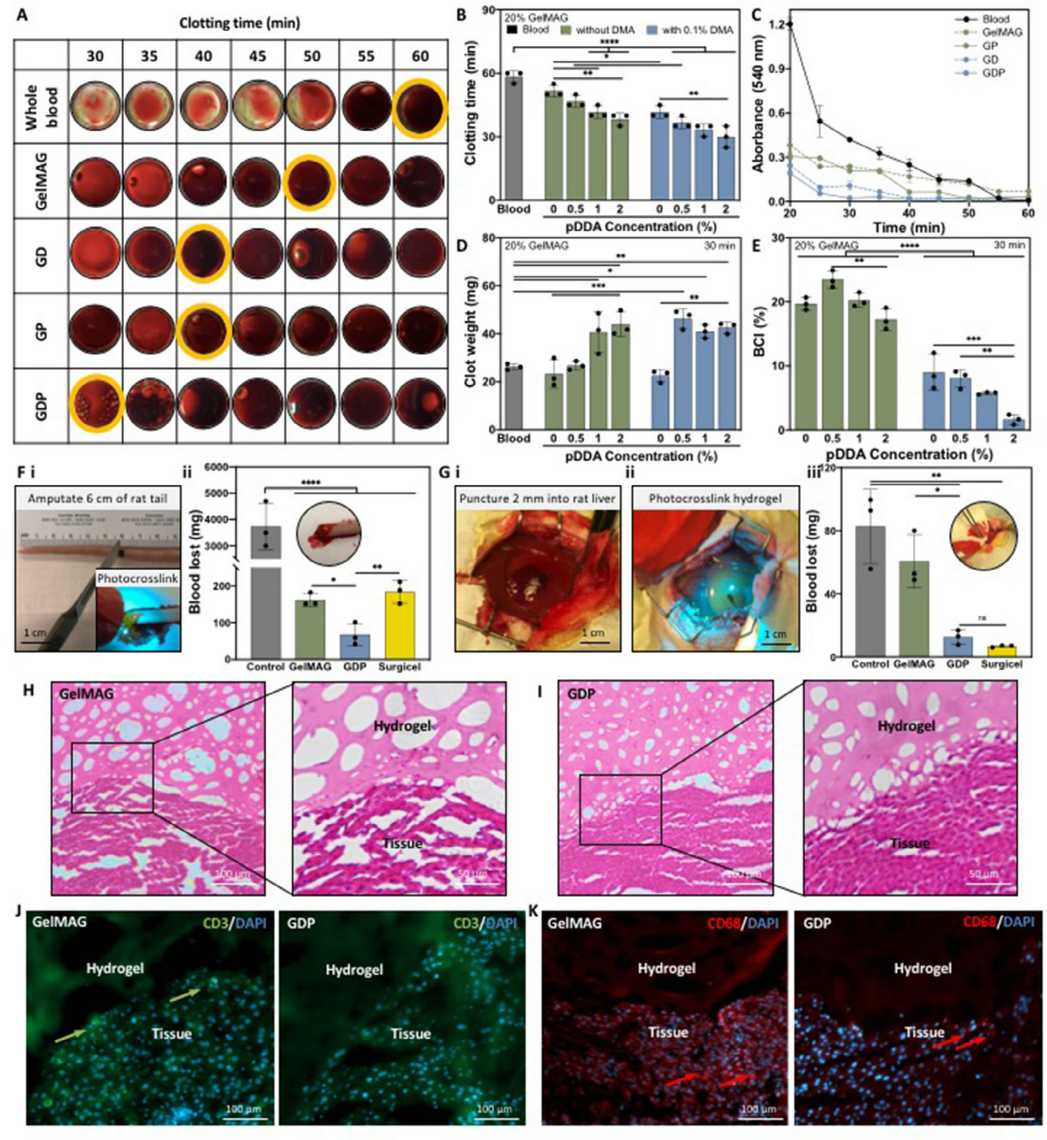
In vitro and in vivo hemostatic properties of GDP hemostatic sealant. In vitro hemostatic properties: A) Hemostatic blood clotting assay performed on citrate‐activated whole blood treated with GelMAG, GD (with 0.1% w/v DMA), GP (with 2% v/v pDDA), and GDP (with 0.1% w/v DMA and 2% v/v pDDA). Whole blood without any hydrogel treatment was used as a control. B) Qualitative evaluation of complete blood clotting time taken by quenching clot formation with saline at specific time points after blood was treated with g GDP hydrogels with varying amounts of DMA and pDDA. Untreated blood was used as a control. C) Quantitative analysis of blood clotting time by measuring hemoglobin absorbance at 540 nm. D) Clot weight for all samples measured 30 min after treatment of activated blood with hemostatic hydrogels. E) Blood clotting index (BCI) of GDP hydrogels prepared with varying amounts of pDDA and DMA. Analysis by two‐way ANOVA with Tukey's post‐hoc multiple comparisons test. In vivo hemostatic properties: F) Tail amputation model where (i) 6 cm of the rat tail was amputated and treated with GelMAG or GDP hydrogels, and (ii) blood loss was collected on filter paper for 10 min and compared to commercial hemostat Surgicel. G) Liver puncture model where (i) the upper right lobe of the rat liver was punctured 2 mm deep using a marked scalpel. (ii) GelMAG or GDP prepolymer solutions were then applied and photocrosslinked to form a hemostatic hydrogel, and (iii) blood loss was collected on filter paper for 10 min and compared to Surgicel. Rats that underwent injuries without hydrogel treatment were used as controls for both models. Analysis by one‐way ANOVA with Tukey's post‐hoc multiple comparisons test. Representative H&E stained images of H) GelMAG and I) GDP hydrogels with surrounding liver tissue on day 14 post‐operation. Representative immunostained images for J) T‐cells (CD3, green) and nuclei (DAPI, blue) or K) macrophages (CD68, red) and nuclei in tissue surrounding GelMAG or GDP hydrogels on post‐operation day 14. Data are represented as mean ± SD. ^*^ *p* < 0.05, ^**^ *p* < 0.01, ^***^ *p* < 0.001, ^****^ *p* < 0.0001. n=3 biological replicates per group.

Heavy hemorrhage must be controlled for physiological recuperation of traumatic injuries and prevention of 50% of post‐traumatic mortality.^[^ ^25^ ^]^ We assessed the in vitro hemostatic activity of the engineered GDP sealant with a standard blood clotting assay using fresh human whole blood. Activated blood was treated with hemostatic hydrogels and, after certain timepoints, the blood clotting process was quenched with saline solution to visually quantify clotting time (**Figure** **5** **A,B**). While whole blood that was not exposed to hydrogels fully clotted in 58 ± 2.7 min, the clotting time was reduced by increasing the concentration of pDDA to 2% (v/v) in both GP (38 ± 2.6 min) or GDP (30 ± 4.4 min) hydrogels (p < 0.0001) (Figure 5B). In order to evaluate the extent of blood clotting after each time point, the concentration of blood cells not entrapped in a clot was measured through ultraviolet‐visible light (UV‐Vis) absorbance of hemoglobin at 540 nm (Figure 5C). The hemoglobin content progressively decreased throughout the assay, which indicated more complete blood clotting. Representative images of clot formation of GDP hydrogels containing 0.5 or 1% (v/v) pDDA (Figure S14, Supporting Information) as well as hemoglobin absorbance after quenching all samples (Figure S15, Supporting Information) confirmed that the presence of both pDDA and DMA endowed the GDP sealant with strong hemostatic properties. After 30 min of exposure to the engineered hydrogels, the blood clots were weighed to evaluate the extent of coagulation with respect to the presence of pDDA (Figure 5D). While the whole blood (control) clot weighed 26 ± 1.4 mg, the clots formed by GP and GDP weighed 39 ± 3.7 mg and 43 ± 2.0 mg, respectively, indicating their advanced onset of hemostasis (p < 0.01). The blood clotting index (BCI) was also calculated based on the amount of unclotted blood after 30 min of hydrogel exposure compared to the untreated control to evaluate the hemostatic performance of the hydrogels. While pure GelMAG had a BCI of 20 ± 0.87%, there was a significant reduction in the BCI of GD (9.0 ± 2.7%) and GDP with 2% (v/v) pDDA (1.6 ± 0.66%) (p < 0.0001) (Figure 5E). It was also found that the BCI was greatly reduced in hydrogels containing both pDDA and DMA, indicating their superior hemostatic ability. Both DMA and pDDA likely play a role in blood coagulation due to their charged functional groups that can form electrostatic interactions with anionic erythrocytes and aggregate them. Furthermore, the charged substances may also facilitate the activation of the intrinsic coagulation cascade through the recruitment of Factor XII and related proteins.^[^ ^27^ ^]^ In addition to their biochemical activity, the engineered hydrogels likely expedite hemostasis due to their high‐water absorption ability. All samples reached their equilibrium swelling ratio after 6 h of incubation in Dulbecco's Phosphate Buffered Saline (DPBS) (Figure S16, Supporting Information). At this point, GelMAG hydrogel had swelled 23 ± 9.4% while GDP sealant swelled 344 ± 34.3% (p < 0.0001). The charge density of the hydrogels containing both pDDA and DMA permitted water infusion into the matrix, which likely helped the hemostatic sealant absorb blood to initiate coagulation.^[^ ^28^ ^]^

We further assessed the in vivo hemostatic efficacy of our engineer hydrogels in multiple rat injury models. In a rat tail amputation model, 6 cm of the tail vein was transected, causing profuse bleeding, before the hemostatic sealants were applied and photocrosslinked over the injury (Figure 5F‐i). The untreated control animals lost 3733 ± 850 mg of blood, which was collected on filter paper for a period of 10 min (Figure 5F‐ii). A commercial hemostatic patch based on oxidized cellulose, Surgicel, was also tested using this model and resulted in 184 ± 20.6 mg blood loss. Compared to this positive control, treatment with GelMAG showed a similar result (162 ± 13.6 mg blood loss) while GDP sealant application caused only 68 ± 21 mg blood loss (p < 0.01). Another model used to test the hemostatic efficacy of GDP was a rat liver puncture model, which is widely reported in literature as a standard for testing hemostatic hydrogels or other biomaterials.^[^ ^29^, ^30^, ^31^ ^]^ Liver injuries can be complicated to treat due to their highly vascularized, soft, and elastic nature. The non‐compressible region also has a high risk of re‐bleeding if inadequately sealed. In the rat liver puncture model, a scalpel was used to puncture 2 mm deep into the liver, and the injury was covered with hydrogels while exsanguination was monitored (Figure 5G‐i, ii). Blood loss in the untreated control group (83 ± 22 mg) was similar to that in the animals treated with pure GelMAG hydrogel (61 ± 11 mg) (Figure 5G‐iii). The use of hemostatic sealant GDP and non‐adhesive hemostat Surgicel resulted in much less blood loss (12 ± 3.9 mg and 6.8 ± 0.14 mg, respectively) (p < 0.01). The GDP sealant accelerated hemostasis not only due to electrostatic interactions between blood and the charged groups on pDDA and DMA, but also because of its strong tissue adhesion, which allows the gel to act as a physical barrier that manages blood loss.^[^ ^28^ ^]^ Therefore, the hemostatic efficacy of GDP was far superior to that of GelMAG for this model.

After the punctured liver was treated, the hemostatic agents were left on the injury for 14 days, during which all rats recovered from the procedure, and no instances of wound dehiscence or internal bleeding were observed. Immunohistochemical (IHC) analysis was performed on the explanted liver tissues after two weeks in order to observe the tissue/sealant interface as well as the host immune response to the hydrogels. Hematoxylin and eosin (H&E) staining on hydrogel/tissue interfaces showed that both GelMAG (Figure 5H) and GDP (Figure 5I) exhibited durable adhesion and retention onto the injury site even after 14 days without causing scarring or abnormal tissue morphology. Immunostaining was also conducted for inflammation‐associated biomarkers like T‐cells (CD3) and macrophages (CD68) to further evaluate the biocompatibility of the hydrogels. Both control GelMAG and GDP hydrogels showed minimal CD3 (marked by green) expression in the tissue surrounding the implants (Figure 5J). Likewise, there was only slight macrophage (marked by red arrows) infiltration into the tissue surrounding either GelMAG or GDP, indicating a negligible immune response to the implanted biomaterials (Figure 5K). Overall, the results proved that the engineered hemostatic sealant could rapidly stop hemorrhage while preventing excessive blood loss and could close the injury for 2 weeks without causing cytotoxicity.

### 2.7 In Vivo Biocompatibility and Biodegradability

**Figure 6:**
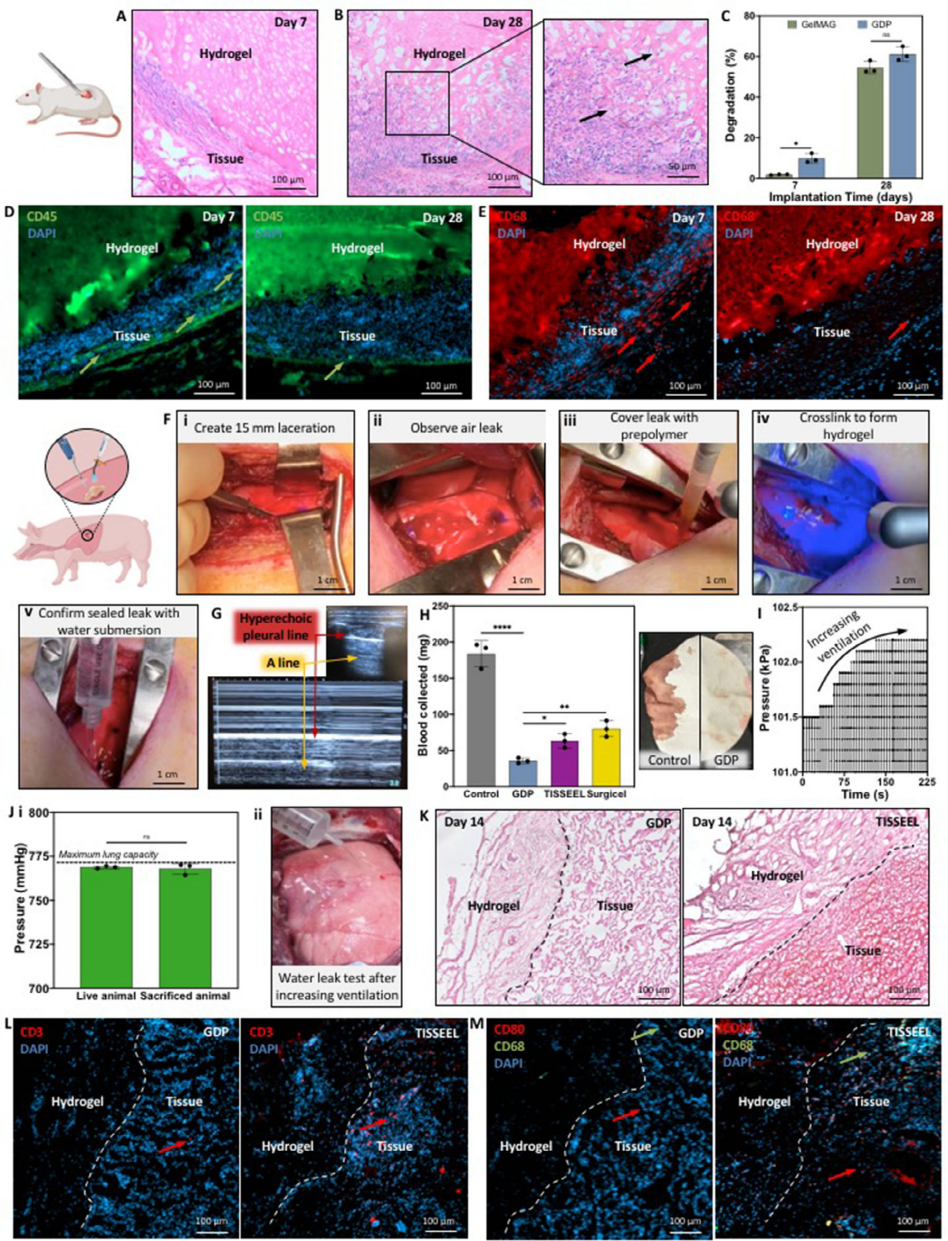
In vivo biocompatibility/biodegradability and sealing/hemostatic efficacy of GDP hemostatic sealant. Rat subcutaneous implantation to evaluate biocompatibility and biodegradability: Representative H&E stainied images of subcutaneously implanted GDP hydrogel into the rat dorsum after A) 7 or B) 28 days of implantation. C) Biodegradation profile of GelMAG and GDP over 28‐day subcutaneous implantation. Analysis by two‐way ANOVA with Tukey's post‐hoc multiple comparisons test. Representative immunostained images of D) hematopoietic cells (CD45, green) and nuclei (DAPI, blue) or E) macrophages (CD68, red) and nuclei in tissue surrounding GDP hydrogels after 7 or 28 days of subcutaneous implantation. Porcine large laceration model to test sealing and hemostatic efficacy: F) Pig lung laceration model to test GDP sealant conducted by (i) creating 15 mm long laceration with a scalpel,^[^ ^38^ ^]^ observing an air leak,^[^ ^38^ ^]^ sealing the injury with GDP precursor that was then (iv) photocrosslinked with visible light to form a hemostatic hydrogel sealant, and (v) confirming that the air leak was sealed by submerging the thoracic cavity in saline and monitoring for air bubbles. G) Thoracic ultrasound taken after the procedure to monitor lung function and signs of pneumothorax. H) In vivo hemostatic test, measuring the amount of blood collected on filter paper for 1 min after application of GDP hydrogel or commercial controls, Surgicel, or TISSEEL over pig lung laceration. Analysis by one‐way ANOVA with Tukey's post‐hoc multiple comparisons test. I) Representative plot of increasing ventilation pressure to pig lungs to monitor hydrogel sealing efficacy after 14 days of implantation. J) Maximum pressure that lacerated lungs sealed by GDP hydrogel could withstand after increasing ventilation pressure to both live and sacrificed animals, and a representative image of syringe administration of saline over the injury to test for air leakage during ex vivo pressure test. K) Representative H&E stained images of GDP or TISSEEL with surrounding lung tissue after 14 days. Representative immunostained images of L) T‐cells (CD3, red) and nuclei (DAPI, blue) or (K) pan‐macrophages (CD68, green), pro‐inflammatory macrophages (CD80), and nuclei in tissue surrounding GDP sealant or TISSEEL on post‐operation day 14. Data are represented as mean ± SD. ^*^ *p* < 0.05, ^**^ *p* < 0.01, ^****^ *p* < 0.0001. n=3 biological replicates per group.

We further evaluated the in vivo biocompatibility and biodegradability of the GDP sealant in a rat subcutaneous implantation model. After the lyophilized gels were implanted into the dorsum for 7 or 28 days, the rats were euthanized so that the explanted tissue could be processed for IHC staining and the implanted hydrogels could be prepared for biodegradation analysis. H&E staining of the hydrogel/tissue interfaces revealed that both GelMAG (Figure S17, Supporting Information) and GDP remained adhered to the subcutaneous tissue for 7 (**Figure** **6**A) and 28 (Figure 6B) days post‐operation. There was no apparent indication of tissue scarring around the gels. Furthermore, there was notable cellular proliferation (shown by the black arrow) into the GDP sealant by day 28, which could enhance prospects for tissue regeneration (Figure 6B). Both hydrogels also showcased biodegradation profiles that could support typical wound healing timelines. The GDP sealant degraded more than GelMAG after 7 days of implantation, possibly due to its higher capacity for water absorbance, but by day 28, both hydrogels underwent similar extents of biodegradation (55 ± 2.9% for GelMAG and 61 ± 3.1% for GDP) (Figure 6C). Immunostaining was also conducted on hydrogel/tissue interfaces to assess the local immune response to the implants. Antibody staining for hematopoietic cells (CD45) revealed that while there was some inflammatory cell activity in the tissue surrounding the GDP hydrogel at day 7, the immune response dramatically decreased by day 28 (Figure 6D). Similarly, there was significant macrophage (CD68) infiltration by day 7 which was reduced by day 28 (Figure 6E). Immunostaining for tissue/GelMAG implants also showed similar trends for immune activity (Figure S18, Supporting Information). The initial inflammation caused by the hydrogels could be due to their constituents. The highly charged pDDA polymer is often used in hydrogels to impart antimicrobial activity since it can disrupt bacterial cell membranes, but this phenomenon can also cause dose‐dependent cytotoxicity.^[^ ^21^ ^]^ Reports note a reduction in cell viability after exposure to 5% (w/v) pDDA.^[^ ^32^ ^]^ Similarly, even though dopamine is naturally occurring, its eventual oxidation and subsequent production of ROS can cause dose‐dependent cytotoxicity.^[^ ^33^ ^]^ Since both pDDA and DMA have limits to their biocompatibility, we confirmed the long‐term biocompatibility and biodegradability of the optimal GDP sealant containing 2% (v/v) pDDA and 0.1% (w/v) DMA. Furthermore, the synthesized hydrogel components can be safely processed by the body after biodegradation. GelMAG is enzymatically degradable, primarily through matrix metalloproteinases, into peptides and amino acids that are biocompatible and safely metabolized or cleared via physiological pathways.^[^ ^34^ ^]^ DMA may be released in small amounts or as oligomers that can be metabolized by enzymes such as monoamine oxidase, and at the concentrations used in our system, the released byproducts are expected to be non‐toxic.^[^ ^35^ ^]^ Lastly, pDDA is a synthetic polymer that may dissociate from the hydrogel matrix as intact chains or short fragments, depending on the degree of erosion. Although high concentrations of pDDA can be cytotoxic, our formulation used low levels, demonstrating biocompatibility.^[^ ^36^ ^]^

### 2.8 In Vivo Efficacy of Biocompatible and Hemostatic GDP Sealant on Pig Lung Lacerations

After evaluating the biocompatibility and efficacy of the GDP hemostatic sealant on small animals, we used a porcine lung injury model to test the hemostatic and sealing capabilities of GDP on larger injuries that are more representative of real clinical burdens. The treatment of lung injuries can be complex due to their vasculature and constant respiratory motion, which can complicate clot stability and wound sealing and also pose a risk of thrombosis or pneumothorax. Once a thoracotomy was conducted to expose the upper right lobe of the lungs, a 15 mm long laceration with roughly 2–4 mm depth was created (Figure 6F‐i), and we immediately observed bleeding and air leaks (Figure 6F‐ii). The injury was covered with prepolymer solution (Figure 6F‐iii) that was photocrosslinked with visible light to form the GDP hydrogel (Figure 6F‐iv). Afterward, we confirmed that the hemostatic sealant could seal the laceration by submerging the thoracic cavity with saline and monitoring for a lack of air bubbles under normal ventilation (Figure 6F‐v). Once the chest cavity was closed, a thoracic ultrasound was performed to assess for signs of wound dehiscence or pneumothorax. The sliding hyperechoic pleural lines indicated the junction of visceral and parietal pleura, while the A‐lines indicated that the lungs were filled with air (Figure 6G). The absence of B‐lines, which signify extravascular lung water (EVLW), also supported the conclusion that the lung laceration had been tightly sealed by the GDP hydrogel.

When the hemostatic and adhesive GDP hydrogel was applied to the laceration, we measured in vivo hemostatic efficacy by collecting the amount of blood lost after wound treatment. Compared to the untreated injury, which lost 184 ± 16.6 mg blood, the wound that was treated with GDP sealant only lost 36 ± 3.4 mg blood (p < 0.0001) (Figure 6H). A non‐adhesive hemostatic agent, Surgicel, and another hemostatic sealant, TISSEEL, were also tested to compare the efficacy of our engineered hydrogel with the commercial products. We noted that treating the lacerations with GDP sealant caused less blood loss compared to both Surgicel (80 ± 10 mg, p < 0.01) and TISEEL (63 ± 8.6 mg, p < 0.05).

After evaluating the hemostatic properties of the hydrogels, the chest cavity was closed as previously mentioned, while GDP and TISSEEL remained in place to seal the laceration for 14 days. The pigs survived this recovery period without loss of body mass (Figure S19A, Supporting Information) or incidences of pneumothorax (Figure S19B, Supporting Information). After 14 days post‐operation, we tested the adhesion of the GDP sealant to the defect by applying ventilator‐driven air pressure to the sealed lungs. We observed gradually increasing pressure in the lungs until their maximum ventilation capacity without signs of burst or failure (Figure 6I). On the live animals, we noted in vivo sealing efficacy since the lungs could undergo high pressures that were within the range of normal lung capacity (Figure 6J). After animal sacrifice, we opened the thoracic cavity and increased ventilation pressure while injecting saline over the injury to monitor the sealing capability of the hydrogel (Figure 6J). We confirmed the *ex vivo* sealing efficacy of the hydrogel since the GDP‐sealed lungs could withstand high pressures. We then explanted the hydrogels with the surrounding tissue for IHC analysis. H&E staining of the interface of GDP and tissue revealed robust adhesion and retention of the hydrogel with no signs of fibrosis, and normal morphology (Figure 6K). On the other hand, while TISSEEL also adhered to the lung tissue, it appeared to cause fibrosis, as seen by the densely packed nature of the tissue surrounding the implant. Fibrin sealants like TISSEEL are known to hinder nutrient diffusion and wound healing, and sometimes even present inflammation‐related safety concerns due to their inclusion of aprotinin.^[^ ^37^ ^]^ To further assess the local immune response to the implanted hemostatic sealants, we conducted immunostaining for inflammation‐associated biomarkers such as T‐cells (CD3), pan‐macrophages (CD68), and pro‐inflammatory macrophages (CD80). In addition, DAPI‐stained nuclei demonstrated cellular infiltration inside both GDP and TISSEEL during the 14‐day implantation, which supports their suitability for tissue regeneration as well as wound closure (Figure 6L,M). Additionally, the GDP sealant appeared to be biocompatible, as indicated by the minimal presence of T cells in the tissue adjacent to the hydrogel (Figure 6L). TISSEEL, on the other hand, caused significant infiltration of T‐cells in the surrounding lung tissue. Similarly, while GDP sealant did not cause much recruitment of pan‐macrophages or pro‐inflammatory macrophages, there was notable CD80 expression in tissue adjacent to TISSEEL (Figure 6M). The heightened host immune response to TISSEEL, seen by fluorescent antibody staining, matched the fibrosis that was observed during histology, emphasizing the potential risks of using fibrin‐based sealant for injury management. Moreover, the results from the porcine lung laceration model proved the efficacy of GDP as a hemostatic and adhesive sealant as well as a biocompatible implant for long‐term wound treatment.

## 3 Discussion

We engineered a hemostatic and antibacterial sealant, GDP, for treating multi‐dimensional injuries on dynamic tissue surfaces. The GDP hydrogel exceeded the fundamental requisites of therapeutics designed for hemorrhaging elastic organ injuries, including tunable and biomimetic physical properties, wet tissue adhesion, rapid control of exsanguination, antibacterial protection, and biocompatibility. By utilizing derivatives of gelatin as a biocompatible and soft hydrogel base, dopamine for wet tissue adhesion, and a quaternary ammonium‐containing polymer for antibacterial and hemostatic properties, the GDP hydrogel was designed to harness multifunctional properties that could ease the clinical burdens often associated with wound management.

In order to permit adequate cellular communication in the damaged tissue that can improve tissue regeneration outcomes, the mechanophysical properties of the GDP hydrogel were characterized. As observed in ^1^H NMR spectra, there was a higher degree of crosslinking in GelMAG hydrogels compared to either GP or GD gels. Both pDDA and DMA likely reduced the amount of covalent bonding between GelMAG methacryloyl groups through steric hindrance (in the case of pDDA) or consumption of methacryloyl residues (in the case of DMA). However, there was a higher degree of crosslinking in the GDP composite since the presence of DMA and pDDA together could support cation‐π interactions and other physical interactions that resulted in improved hydrogel cohesion. The interactions between pDDA and DMA might also reduce the radical scavenging effect of DMA that would normally obstruct photopolymerization.^[^ ^39^ ^]^ Our previous study involved chemical conjugation of dopamine to gelatin followed by gelatin methacrylation to yield GelMA‐catechol (GelMAC), which exhibited lower degree of crosslinking (65%) compared to pure GelMA (90%).^[^ ^40^ ^]^ Similarly, in another study, a drop in mechanical properties (storage modulus) was observed after dopamine derivatives were added to gelatin‐based hydrogels, substantiating their decreased crosslinking density.^[^ ^41^, ^42^ ^]^ Other studies overcame the dopamine‐induced mechanical weakening through the incorporation of minerals or nanoclays.^[^ ^43^, ^44^ ^]^ In this work, we avoided inadequate crosslinking by manipulating the interactions between each component in the 3D network of the GDP hydrogel to achieve a crosslinking density of 87% without the need for adding any inorganic materials.

Compared to the pure GelMAG hydrogel, the GD composite exhibited lower tensile Young's modulus and strength, likely as a result of the reduced crosslinking density. However, the increased stretchability and toughness could be attributed to the plentiful non‐covalent interactions, such as hydrogen bonding and *π–π* stacking, that are instigated by the catechol region of DMA and which permitted energy dissipation upon deformation. Compared to GelMAG or GD hydrogels, the GP and GDP composites containing low concentrations of pDDA exhibited lower toughness and tensile strength, which could also be due to their obstruction of methacryloyl interactions that resulted in low crosslinking density. Increasing pDDA concentration, however, bolstered the mechanical properties since there was more potential for physical crosslinking, including electrostatic and cation‐π interactions. Therefore, the GDP sealant that contained 0.1% (w/v) DMA and 2% (v/v) pDDA was selected as an optimal formulation since it could provide biologically relevant mechanical properties to support native soft tissues.

Mechanical mismatch is a limitation for many tissue sealants and hemostatic biomaterials. For example, a commercial pleural air leak sealant, Progel, claimed to exhibit high elasticity to support lung expansion, but it only had 40 kPa ultimate strength and 25% stretchability, which could be inadequate for treating injuries on more elastic organs.^[^ ^17^ ^]^ Similarly, commercial sealants like Coseal and hemostats like BioGlue were characterized with high stiffness (100 kPa for Coseal and 3122 kPa for BioGlue) and low extensibility (5–10%).^[^ ^45^, ^46^ ^]^ Furthermore, many tissue sealants utilizing catechol‐containing compounds to enhance adhesion properties may also render mechanical properties that could be unsuitable for soft, dynamic tissues like the lungs. For example, dopamine‐conjugated GelMA that was photocrosslinked with visible light and then chemically crosslinked with ferric ions exhibited a Young's modulus in the range of 300–400 kPa.^[^ ^29^ ^]^ Another GelMA‐based hydrogel with intercalated DMA oligomers exhibited compression modulus and tensile toughness in the MPa range, resulting in a very stiff gel.^[^ ^47^ ^]^ Even GelMA hydrogels with tannic acid had a high tensile Young's modulus of ≈200 kPa, which could be too stiff for many soft tissues like lungs.^[^ ^48^ ^]^ Our previous work on polydopamine‐modified GelMA exhibited low Young's modulus (5–30 kPa) and high extensibility (140%) that could be suitable for elastic organ repair, but it had very low adhesion strength (0.3–4 kPa).^[^ ^42^ ^]^ In another work, a hydrogel containing GelMA and DA‐modified HA also exhibited low Young's modulus (17 kPa) and high extensibility (100%), but it had a swelling ratio in the range of 1000–2000%, which could decrease mechanical strength or increase potential for undesired tissue compression after sweling.^[^ ^49^ ^]^ Our engineered GDP sealant was successfully tuned to foster specific chemical interactions between catechol and quaternary ammonium groups, which produced a soft, tough, and stretchable hydrogel.

The adhesive properties of the GDP sealant were evaluated to assess its suitability for elastic organ injuries. The in vitro adhesion strength of GDP was improved upon the addition of DMA since it is a derivative of L‐DOPA, a chemical responsible for the durable underwater adhesion of marine mussels.^[^ ^50^ ^]^ The catechol groups on DMA or quinone groups on oxidized DMA could facilitate Michael addition or Schiff base interactions with amino groups on the tissue surfaces. The addition of pDDA further enhanced the tissue adhesion capabilities of the GDP sealant due to the potential for electrostatic interactions that can occur with anionic tissue residues. Furthermore, the substantial π‐conjugated electron density of the DMA aromatic ring could strengthen its cation‐π bonding with pDDA quaternary ammonium, which ultimately frees up the catechol alcohol groups for tissue engagement.^[^ ^4^ ^]^

The robust tissue adhesion properties displayed by the GDP sealants far surpassed not only the commercial controls, Coseal and Evicel, but also other biomaterials engineered with catechol‐containing groups for wound closure. For example, dopamine‐conjugated GelMA that was dual‐crosslinked with hydrogen peroxide and ferric ions exhibited ≈15 kPa adhesion strength.^[^ ^51^ ^]^ Another sealant based on the conjugation of DA onto HA showed only 10 kPa adhesion strength.^[^ ^52^ ^]^ Dopamine was also polymerized to form polydopamine and then combined with polyacrylic acid (PAA), but the copolymer displayed at most 17 kPa adhesion strength.^[^ ^53^ ^]^ This adhesion strength was achieved with high concentrations of catechol, which ultimately lowered the Young's modulus of the bioadhesive. Similarly, a glue based on dopamine‐functionalized hyperbranched polymers was prepared for wet tissue adhesion, but it only exhibited ≈12 kPa adhesion strength.^[^ ^54^ ^]^ We previously engineered a polydopamine‐conjugated gelatin hydrogel that was chemically crosslinked with sodium periodate, but that exhibited under 9 kPa adhesion strength.^[^ ^55^ ^]^ On the other hand, dopamine was used to functionalize GelMA or methacryloyl‐modified alginate, which seemed to increase the adhesion strength to 25–35 kPa in the resulting pleural and tracheal sealants.^[^ ^56^ ^]^ A previously described GelMA and DA‐conjugated HA hydrogel with suitable mechanical properties for soft and elastic tissue exhibited high adhesion strength (35 kPa), but also had a very high swelling ratio that could negatively impact long‐term tissue adhesion.^[^ ^49^ ^]^ Our previous DA‐modified GelMA hydrogel exhibited 33 kPa adhesion strength, but it also had a high Young's modulus of ≈300 kPa.^[^ ^29^ ^]^ Similarly, a GelMA and tannic acid‐based bioadhesive reported 80 kPa adhesion strength as well as 175 kPa Young's modulus, which could be too stiff for many soft tissues.^[^ ^48^ ^]^

Our previous studies have also tested the ex vivo sealing efficacy of biopolymer‐based sealants on 10 mm wide shallow pleural defects on pig lungs.^[^ ^17^, ^57^ ^]^ For example, our previously described catechol‐modified GelMA hydrogel was complexed with ferric ions, displayed an ex vivo burst pressure of 1.8 ± 0.14 kPa on a 10 mm pleural defect.^[^ ^57^ ^]^ After it was combined with poly(ethylene glycol) diacrylate (PEGDA), the burst pressure slightly increased to 1.9 ± 0.31 kPa. Similarly, our previously engineered MeTro sealant displayed 2.9 ± 0.49 kPa burst pressure for this type of injury.^[^ ^17^ ^]^ Meanwhile, GDP sealant exhibited higher burst pressures not only on shallow pleural defects but also on large, multi‐dimensional injuries created on highly elastic and pressurized organs, thereby showcasing its potential for clinical translation. Overall, most medical adhesives with low adhesion properties may be suitable for some instances of wound closure, but their efficacy on elastic and dynamic organs could be highly variable. In our engineered GDP sealant, pDDA could engage with the aromatic group of DMA through cation‐π interactions, which could potentially reduce intramolecular hydrogen bonding between hydrogel moieties and DMA and free up the adhesive alcohol residues for tissue engagement.^[^ ^58^ ^]^ Therefore, through careful manipulation of the chemical microenvironment, we successfully maximized the adhesive properties of the GDP sealant.

In addition to the required mechanical and adhesion properties for treating elastic organ injuries, our GDP sealant provided both antibacterial and hemostatic abilities for better clinical translation. Antiseptic wound dressings have been designed using iodine (e.g., Iodoflex),^[^ ^59^ ^]^ silver nanocrystals (e.g., Acticoat),^[^ ^60^ ^]^ inorganic materials (e.g., GO),^[^ ^61^ ^]^ or even charged biopolymers (e.g., chitosan).^[^ ^62^ ^]^ Even though many of these materials have successfully prevented bacterial proliferation, their high charge or ability to generate ROS may cause cytotoxicity, which could hinder wound healing. For example, a hydrogel based on DA, chitosan, and acrylamide for hemostatic and antibacterial properties depicted high adhesion strength (35 kPa) but demonstrated only short‐term biocompatibility and had a complicated material synthesis.^[^ ^63^ ^]^ An antibacterial GelMA and adenine acrylate releasing copper ions was shown to have biocompatibility issues (lower viability compared to controls) as well as low adhesion strength under 10 kPa.^[^ ^64^ ^]^ Another hydrogel based on pDDA‐functionalized bacterial cellulose, PDA, and polyacrylamide was developed as an antibacterial wound dressing, but it exhibited ≈16 kPa adhesion strength.^[^ ^65^ ^]^ On the other hand, the GDP sealant exhibited rapid antibacterial properties that lasted for a longer duration without compromising its other essential wound healing properties. GDP's contact activation of microbial resistance likely stemmed from the adsorptive and swelling properties of the porous hydrogel that allowed polycationic pDDA to diffuse out of the gel and disrupt the bacterial membrane, thereby impeding proliferation.^[^ ^66^, ^67^ ^]^

Hemostats with weak adhesive abilities can prove fatal. Previously, surgical hemostats Surgicel and Oxycel were used to control bleeding during thoracotomy procedures, but they were left in situ and later found detached from the injury site, causing severe medical complications.^[^ ^68^ ^]^ Do to their low tissue adhesion, the hemostats migrated to the spinal cord and caused paraplegia in multiple patients. To overcome this problem, hemostatic patches were developed with adhesive properties, but many require mechanical pressure to adhere to the injury.^[^ ^69^ ^]^ In doing so, they can cause damage to delicate soft tissue (e.g., blood vessels) and to non‐compressible trauma (e.g., head, neck, torso). Hemostatic bioadhesives have also been developed, such as one based on DA‐grafted GelMA, chitosan, and glycerin that had high adhesion strength, but also had low extensibility in the range of 40–60% and BCI in the range of 25–60%.^[^ ^7^ ^]^ While some compositions exhibited low BCI, indicating faster blood clotting time, they also had potential cytotoxicity concerns due to the use of UV light for hydrogel formation. Another DA‐containing hemostatic glue exhibited a 65% BCI, but had low tissue adhesion strength under 12 kPa.^[^ ^54^ ^]^ In this work, the GDP hemostatic sealant exhibited a 2% BCI, which highlighted its potential for rapid and effective hemostasis.

Other hemostatic hydrogels under development were also tested in rat tail and liver injury models. For example, a conductive and hemostatic hydrogel based on DA‐grafted gelatin and GO decreased the amount of blood lost after liver hemorrhage by 80% compared to the untreated control.^[^ ^30^ ^]^ However, the hydrogel had relatively low tensile and adhesion strength (both ≈15 kPa). Furthermore, GO has known cytotoxicity concerns due to its potential to cause oxidative stress and DNA damage.^[^ ^70^ ^]^ In our previous study on hemostatic hydrogels based on DA‐modified GelMA and ferric ions, we were only able to prevent 48% blood loss after liver puncture.^[^ ^40^ ^]^ Another hydrogel designed with antibacterial and hemostatic properties based on chitin and gold nanoparticle‐filled halloysite nanotubes (Au@HNT) decreased only 55% of blood loss after liver injury and 61% of blood loss after tail amputation.^[^ ^71^ ^]^ Hemostatic biomaterials often employ natural polymers like chitosan to accelerate blood clotting. To that end, a hydrogel engineered with carboxymethyl chitosan, dextran, and polyglutamic acid prevented 69% of blood loss from a tail amputation.^[^ ^72^ ^]^ Nevertheless, the efficacy of the hydrogel could be limited in various surgical settings due to its low extensibility of 2.5%. In another study, a hydrogel based on chitosan, silk, and Pluronic F127 prevented 82% of tail blood loss.^[^ ^73^ ^]^ However, this hemostat required 16 h of soaking in tannic acid solution in order to achieve sufficient tissue adhesion strength (15 kPa), which could be difficult to perform in surgical settings. Herein, the engineered GDP adhesive could prevent 86% of blood loss after liver puncture and 98% of bleeding after tail amputation, which was more effective than other developed hemostatic biomaterials.

The hemostatic and sealing efficacy of the engineered GDP hydrogel was ultimately proven on large lacerations conducted on pig lungs. The GDP sealant caused rapid hemostasis and minimal blood loss compared to two commercial hemostats: Surgicel and TISSEEL. The GDP sealant also exhibited long‐term sealing of the pig lung laceration and high biocompatibility, especially compared to the hemostatic sealant TISSEEL. While the fibrin‐based sealant could have hindered nutrient diffusion that resulted in tissue fibrosis,^[^ ^37^ ^]^ the GDP hydrogel permitted normal tissue regeneration without instances of fibrosis or severe immune reaction. Overall, we demonstrated the efficacy of the GDP hemostatic sealant as a cost‐effective, scalable, and multifunctional treatment for elastic organ injuries.

For potential clinical translation, the individual components of GDP can be lyophilized and stored at 4 °C, with an estimated stability exceeding 1–2 years based on the component shelf lives. These can be reconstituted with sterile photoinitiator solution immediately prior to use. The prepolymer solutions may be stored refrigerated for short‐term use (within 1‐4 weeks), allowing flexibility for clinical workflows. Together, these considerations suggest that the hydrogel system could be feasibly adopted for point‐of‐care use with straightforward sterilization, storage, and application protocols.

Despite the promising results of this study, several limitations must be acknowledged. One shortcoming of the GDP hydrogel could be its necessity for photopolymerization. Although in situ photocrosslinking can control crosslinking dynamics and tune the mechanophysical properties of the hydrogel, its application is only feasible on open and accessible wounds. Thus, our future work can investigate crosslinking methods that are more suitable for minimally invasive procedures. Additionally, while the hydrogel exhibited potent in vitro antibacterial properties, it would be valuable to study its response to other strains of antibiotic‐resistant bacteria as well as to assess its in vivo bacterial resistance on internal and external injuries with increased risk of biofilm formation. Furthermore, while the biodegradation profile of the engineered sealant was characterized over 28 days, we did not observe complete biodegradation which should be assessed in the future. Furthermore, although the porcine lung incision model was conducted over a two‐week timeframe, it confirmed the early hemostatic and sealing efficacy of GDP. Nevertheless, long‐term in vivo biodegradation studies as well as in‐depth analysis of the safety and clearance of the degradation byproducts of GDP should be conducted. We can also increase the biological study size for a more comprehensive evaluation of GDP's statistical efficacy. These limitations do not reduce the importance of this study. Rather, they underscore key areas for future research to improve the safety, efficacy, and clinical relevance of the GDP hemostatic sealant.

## 4 Experimental Section

### Study Design

This study details the performance of an antibacterial and hemostatic bioadhesive based on GelMAG, DMA, and pDDA. A comprehensive evaluation was conducted, including physical, in vitro, ex vivo, and in vivo experiments, which were conducted to characterize the engineered hydrogels and evaluatetheir safety, antibacterial, and hemostatic properties. Prior literature and power analysis were used to determine sample size and to ensure statistical validity while minimizing animal use in compliance with ethical standards. To enhance reliability, all experiments were conducted in triplicate. In vivo studies incorporated randomization of experimental groups as well as blinding during outcome analysis to reduce selection bias. Data collection was stopped at pre‐specified timepoints or when pre‐determined criteria were met, including mechanical failure, clot formation, or survival periods. Data inclusion criteria ensured that only samples that met experimental conditions without contamination or procedural errors were analyzed. Exclusion criteria accounted for procedural inconsistencies.

### Materials

Gelatin from porcine skin (Gel strength 300, Type A), methacrylic anhydride (MA), GMA, pDDA, Eosin Y, TEA, type II collagenase, ethyl acetate, and hexane were purchased from Sigma Aldrich. Dopamine hydrochloride, VC, DPBS, PrestoBlue reagent, and Abcam Anti‐CD68 antibody were purchased from ThermoFischer Scientific. DMEM, *P. aeruginosa* (catalog number 27853, strain Boston 41504), and *MRSA* (catalog number 33591, strain 328) were purchased from ATCC, fetal bovine serum (FBS) was purchased from Corning, and penicillin/streptomycin was purchased from Life Technologies. Commercial live/dead kits (calcein AM and ethidium homodimer), AlexaFlour 594 (phalloidin), and DAPI were purchased from Invitrogen. Mayer's hematoxylin was purchased from Electron Microscopy Sciences. ^1^H NMR solvents deuterated dimethyl sulfoxide (DMSO‐d6) and deuterium oxide (D2O) were purchased from Fischer Scientific.

### Synthesis of GelMAG

Gelatin was chemically modified with GMA to produce GelMAG. First, 10% (w/v) porcine gelatin was dissolved in DPBS at 60 °C while under constant, vigorous stirring. Once fully dissolved, 0.16% (v/v) GMA was added dropwise at 60 °C under stirred conditions and left for 4 h. The mixture was then diluted (2X) with DPBS to quench the methacrylation. Afterward, the solution was dialyzed using 12–14 kDa MWCO dialysis tubing for 7 days against 50 °C deionized water to remove impurities like salts and unreacted GMA. The clear dialyzed solution was frozen at −80 °C overnight and then lyophilized for 7 days until a white foam‐like solid was produced. GelMAG was stored at 4 °C.

### Synthesis of DMA

DA was chemically modified with MA to produce DMA using a modified, previously reported synthesis method.^[^ ^47^ ^]^ Briefly, 5% (w/v) dopamine hydrochloride in a 5:2 mixture of borax and sodium bicarbonate solution was prepared under nitrogenated conditions at room temperature. Then, 25% (v/v) MA tetrahydrofuran was added dropwise to the mixture. During the reaction, the pH was maintained above 8 by adjustment with sodium hydroxide. The reaction continued overnight and was then washed in triplicate with ethyl acetate. Afterward, the pH of the aqueous phase was reduced to below 2 by adjustment with hydrochloric acid, and the organic layer was separated, concentrated by rotary evaporator, and mixed with cool hexane to precipitate DMA. The precipitated DMA was further purified in cooled hexane, dried under vacuum conditions, and stored at 4 °C.

### Preparation of the GDP Sealant

Hydrogels were prepared by dissolving 20% (w/v) GelMAG in a photoinitiator solution of 0.08% (w/v) Eosin Y, 0.9% (v/v) TEA, and 0.9% (w/v) VC at 37 °C. Once fully dissolved, 0.1% (w/v) DMA was added, mixed, and incubated overnight to conjugate DMA onto the methacryloyl residues of GelMAG. Next, pDDA was added at concentrations of 0.5, 1, or 2% (v/v) and quickly mixed to ensure homogenous distribution of the polyelectrolyte. Prepolymer solutions were crosslinked in a polydimethylsiloxane (PDMS) mold for 4 min with visible light (450–550 nm) using an LS1000 Focal Seal Xenon Light Source (100 mW cm^−2^, Genzyme).

### 4.1 ^1^H NMR Spectroscopy

^1^H NMR analysis was conducted on prepolymer solutions and hydrogels to calculate the degree of methacrylation (DM) and the degree of crosslinking.^[^ ^74^ ^]^ Samples were frozen in −80 °C overnight, lyophilized for 2 days, and then fully dissolved at a concentration of 10 mg/mL in deuterated dimethyl sulfoxide (DMSO‐d6). The spectra were obtained using a 400 MHz Bruker AV400 spectrometer (64 scans). All spectra were processed with phase and baseline corrections and assigned a reference point at the residual singlet peak of DMSO‐d6 at 2.54 ppm before analysis. The DM was calculated by finding the ratio of amine protons on GelMAG lysine residues to the amine protons in gelatin. The two vinylic protons residing on the methacryloyl group of GelMAG gave rise to two separate singlet peaks at 5.74 and 6.13 ppm. The peaks at 2.78 ppm corresponding to the primary amine protons in gelatin and the secondary amine protons in GelMAG were integrated to determine DM according to Equation 1.

(1) $$\mathrm{DM}\left(\%\right)=\left(1-\frac{I_{2^{\circ }\mathrm{amin}\mathrm{epro}\mathrm{tons}\;\mathrm{on}\;\mathrm{GelM}\mathrm{AG}}}{I_{1^{\circ }\mathrm{amin}\mathrm{epro}\mathrm{tons}\;\mathrm{on}\;\mathrm{gela}\mathrm{tin}}}\right)*100$$

^1^H NMR analysis was conducted on dopamine hydrochloride and DMA with the same methods for sample preparation and spectra processing. The methacryloyl protons on DMA gave rise to two peaks at 5.33 and 5.64 ppm. ^1^H NMR analysis was also conducted on GDP precursors and hydrogels with various amounts of DMA and pDDA to calculate the different DCs using Equation 2.

(2) $$DC\left(\%\right)=\left(1-\;\frac{I_{methacryloyl\;protons\;in\;hydrogel}}{I_{methacryloyl\;protons\;in\;precursor\;solution}}\right)*100$$

### FTIR Analysis

FTIR analysis was conducted in transmittance mode using a Thermo Scientific Nicolet 8700 spectrometer. Hydrogel samples were lyophilized and then analyzed at room temperature. Spectra were collected in the range of 4000–500 cm^−1^ with a resolution of 4 cm^−1^ and 32 scans per sample. Hydrogels tested were pure GelMAG, GP (with 2% v/v pDDA), GD (with 0.1% w/v DMA), and GDP (with 0.1% w/v DMA and with 2% v/v pDDA).

### Rheological Testing

Rheological properties were measured using an Anton Paar MCR302 rheometer. The rheometer had a sandblasted plate top geometry (8 mm) and a Peltier bottom place that remained at 25 °C throughout the experiments. Gelation characteristics were analyzed by monitoring the storage and loss moduli over a period of 10 min (time sweep experiment) where the prepolymer solution was photocrosslinked with visible light for 4 min. Hydrogel stability was assessed with a frequency sweep over a range of 0.1 to 10 1/s, performed at a consistent strain of 0.1%. The linear viscoelastic region was measured using a strain sweep ranging from 0.01 to 100% strain.

### SEM Imaging

SEM images were taken of hydrogel surfaces after bacterial inoculation and from the hydrogel/tissue interfaces. Hydrogels were washed thrice with DPBS, fixed in 2.5% (v/v) glutaraldehyde and 4% (v/v) paraformaldehyde, washed again, and then serially dehydrated in ethanol (30% to 100% (v/v)). The hydrogels then underwent critical drying (Tousimis Autosamdri‐810 Critical Point Dryer), gold sputtering (Denton Bench Top Turbo‐IV Evaporator), and SEM imaging (ZEISS Supra 40VP SEM).

### Zeta Potential Test

Zeta potential measurements of 50 µg mL^−1^ prepolymer solutions in DI water were taken at 25 °C using universal DTS1070 folded capillary cells, Malvern Zetasizer Nano‐Z equipment, and a DTS (Nano) software (version 4.20).

### Conductivity Test

Hydrogel conductivities were measured after photocrosslinking and submerging in DI water, which facilitated ionic conductance. The hydrogels were placed between two gold nanochips, and conductivity was measured with a CorrWare potentiostat software for electrochemical analysis (−0.4 OC to 0.4 OC, 50 mV S^−1^). The slope of the electrical current and voltage data determined the conductivity.

### Swelling Profile

Swelling ratios were obtained by incubating hydrogels in DPBS at 37 °C for 48 h. The dry mass (W_o_) and mass taken at pre‐determined time points (W_i_) were used in Equation 3 to assess the swelling profile.

(3) $$Swelling\;ratio\left(\%\right)=\left(\frac{W_{i}-\;W_{o}}{W_{o}}\right)*100$$

### Mechanical Characterization

For tensile tests, 80 µL of precursor solutions were photocrosslinked to prepare hydrogels in a rectangular polydimethylsiloxane (PDMS) mold (8 mm length, 5 mm width, 1 mm depth). Dimensions were confirmed using a digital caliper, and then hydrogels were secured in tensile tape, placed in an Instron 5943 mechanical tester, and pulled to failure at a strain rate of 1 mm/min while data were collected on Bluehill Universal software. Ultimate strength and stretchability were recorded at the stress and strain, respectively, at failure. Young's modulus was defined as the slope of the initial linear portion of the stress–strain curve at 6–10% of maximum strain. Toughness was measured as the area under the stress–strain curve.

For compression tests, 80 µL of precursor solutions were photocrosslinked to prepare hydrogels in a cylindrical PDMS mold (5 mm diameter, 2 mm depth), where dimensions were confirmed with a digital caliper. The hydrogels were placed between Instron compression plates and compressed to failure at a rate of 1 mm/min while data were recorded using Bluehill Universal software. Compressive modulus was taken as the initial linear slope of the stress–strain curve at 20–30% strain. Cyclic compression testing was separately performed by compressing the hydrogels to 50% strain at a rate of 1 mm/min for 12 cycles. Energy loss was measured from the last cycle using Equation 4, where the loading curve represented the compressed sample and the unloading curve represented the decompressed sample.

(4) $$Energy\;loss\;\left(\%\right)=\left(\frac{Area_{loading\;curve}-A_{unloading\;curve}}{Area_{loading\;curve}}\right)*100$$

### In Vitro Wound Closure

Wound closure tests based on ASTM F2458 were conducted with some modifications. Briefly, porcine skin tissue was rid of hair and fat, cut into rectangular pieces (3 cm x 1 cm), and secured with superglue to glass slides. At the junction of two tissue pieces, 100 µL of prepolymer solution was applied in a square (1 cm x 1 cm) and photocrosslinked to prepare hydrogels. The glass slides were fixed to an Instron 5943 mechanical tester, where they were pulled to failure at a rate of 1 mm min^−1^ while data were collected using Bluehill Universal software. Adhesion strength data were collected at maximum stress, and adhesion energy was taken as the area under the force versus displacement curve.

### Burst Pressure Test

Burst pressure tests based on ASTM F2054 were conducted with some modifications. A custom‐built burst pressure device was assembled using a steel base, top holder, syringe pump, pressure sensor, and a computer with a Pasco Capstone data collecting software. A dry collagen sheet was dampened in water and dried thoroughly with a Kimwipe. The collagen sheet was secured in the holder, and a puncture (diameter: 2 mm) was made in the center of the collagen sheet. Then, 60 µL of prepolymer solutions were applied and photocured to form a hydrogel. The sealed sheet was then pressurized with 10 mL min^−1^ of air, and burst pressure data were collected.

### Ex Vivo Burst Pressure on Ventilated Pig Lung

Burst pressure tests were conducted on ventilator‐assisted pig lungs. The lung was connected to a Veterinary Anesthesia Ventilator (Hallowell EMC) that regulated compressed air at a respiratory rate of 12 bpm and a maximum working pressure of 60 cmH_2_O. The ventilator was connected to a pressure sensor and a computer with the Pasco Capstone software. Deep lacerations with 2.5 mm depth and either 10, 15, or 20 mm length were prepared and sealed with hydrogels. Similarly, both shallow pleural defects (0.5 mm depth and 10 mm diameter) as well as deep punctures with 1.5 mm depth and either 10, 15, or 20 mm diameter were prepared and sealed with hydrogels. Respiratory volume was steadily increased until an air leak was detected by submersion of the lung into a water bath, indicating the burst hydrogel.

### Bacteria Survival and Zone of Inhibition

Two strains of bacteria (*P. aeruginosa* and MRSA) were cultured. Bacterial broths were prepared by inoculating one colony of each strain into its respective culture media and incubating overnight at 35 °C. Bacteria density and viability were assessed with OD readings at 625 nm using a Biotek Eon Microplate Spectrophotometer. To start the antibacterial assay, both bacterial broths were diluted to an OD 0.06, treated with sterile hydrogels, and incubated. Bacteria concentration was measured through OD measurements and also using a spread plate method, involving plating the diluted supernatant from the treated bacterial cultures, incubating overnight at 35 °C, and then counting CFU. Concentration (CFU/mL) was calculated based on the dilution factor and volume plated. Bacterial survival rate and log reduction were measured based on the concentration of treated bacterial samples. To measure ZOI, bacterial suspensions at an OD 0.06 were evenly spread onto agar plates. Cylindrical hydrogels (diameter: 5 mm, height: 2 mm) were prepared, submerged in DPBS for 1 h, lightly dried, placed onto the agar plate, and incubated overnight at 35 °C. The following day, the ZOI between the edge of the hydrogel and the start of the bacteria coverage was measured using a digital caliper. MIC was determined by preparing serial dilutions of the antibacterial materials. For ciprofloxacin, the solution was added to a 24 well‐plate containing bacteria, whereas for GDP hydrogels, different volumes of precursor were crosslinked to form hydrogels that were sterilized and placed into 24 well‐plates. OD measurements after 1day of incubation indicated the MIC based on which concentration prevented bacterial growth. Bacteria staining was conducted using a Live/Dead Bac Light Bacterial Viability kit (Invitrogen) following the manufacturer's protocol, and imaging was done AxioObserver Z1 inverted microscope (Zeiss).

### In Vitro Hemostatic Test

In vitro hemostatic tests were conducted with human blood with the approval of the UCLA Institutional Biosafety Committee (IBC) under the Biological Use Authorization (BUA)‐2018‐116‐001. Hydrogels were prepared at the bottom of a well plate and prewarmed for 5 min before adding human fresh whole blood from a diabetic patient (Zen‐Bio) activated with 0.1 m calcium chloride (9:1). At specific time points, blood clotting was quenched with saline solution, and liquid was removed to assess clot formation and qualitatively determine clotting time. Simultaneously, hemoglobin absorbance at 540 nm was measured from the extracted liquid using a Nanodrop One/One^C^ Microvolume UV–vis Spectrophotometer (ThermoFischer). A baseline correction at 750 nm was included for the entire spectrum. BCI was determined using Equation 5 at the timepoint when the fast clotting sample had fully clotted. Here, A represents hemoglobin absorbance. Clotting weight was measured at this same timepoint.

(5) $$BCI\left(\%\right)=\frac{A_{sample}}{A_{whole\;blood\;control}}*100$$

### In Vitro Biocompatibility Tests

In vitro biocompatibility studies were conducted using NIH3T3 fibroblast cells (catalog number CRL‐1658, 3T3‐ Swiss albino cell line, ATCC). Cells were cultured in Dulbecco's Modified Eagle's Medium (DMEM, ATCC) supplemented with 10% FBS and 1% penicillin/streptomycin antibiotics. NIH3T3 were cultured on a Falcon polystyrene tissue culture flask with a vented cap (Corning) and incubated at 37 °C with 5% CO_2_ infusion. After confluency, cells were seeded at a density of 1 x 10^4^ cells mL^−1^ into 24‐well plates and incubated with GelMAG or GDP hydrogels that were sterilely prepared and loaded into transwell inserts. Sterile hydrogels were prepared by UV sterilizing all constituents (GelMAG polymer, DMA powder, and pDDA solution) and purifying PBS and photoinitiator solution using a 0.22 µm sterile filter. Then, the components were combined, the prepolymer was photocrosslinked to form hydrogels, and the hydrogels were UV sterilized before they were loaded into transwell inserts.

Viability of NIH3T3 cells was measured using a live/dead kit (Invitrogen) according to the manufacturer's instructions. Briefly, cells were stained with 0.05% Calcein‐AM and 0.2% ethidium homodimer‐1 in DPBS for 20 min at 37 °C. Fluorescent imaging was performed after 1, 3, and 5 days of cell culture using an AxioObserver Z1 inverted microscope (Zeiss). Live cells appeared green, and apoptotic cells appeared red. Cell viability was determined by dividing the number of live cells by the total cell count.

Spreading and morphology of the NIH3T3 cells were observed by conducting Actin/DAPI staining. Cells were first fixed with 4% (v/v) paraformaldehyde (10 min), washed with 0.5% (v/v) Triton‐X (10 min), and then blocked with 1% (v/v) BSA (30 min). Afterward, cells were stained with 0.1% (v/v) Phalloidin and 0.05% (v/v) DAPI in DPBS (10 min), washed three times with DPBS, and then imaged using an AxioObserver Z1 inverted microscope (Zeiss). Actin filaments were stained green, and nuclei were stained blue. Cell number was quantified by positively stained F‐actin per unit area.

Metabolic activity was quantified using a PrestoBlue assay (Life Technologies). Cells were incubated with 10% (v/v) PrestoBlue solution in DMEM for 45 min at 37 °C. Then, fluorescence was measured at 600 nm using a Synergy LX Multi‐mode Reader.

### In Vivo Biocompatibility, Biodegradation, and Hemostatic Efficacy Using Rat Models

Animal studies were approved by IACUC (ARC‐2021‐113) at the University of California at Los Angeles (UCLA). Male Wistar rats (≈200 g) were purchased from Charles River Laboratories (Boston, MA). Rats were given anesthesia by inhalation of 4% isoflurane, which was maintained at 1.5% during surgery. In vivo biocompatibility and biodegradability were assessed using a rat subcutaneous implantation model. First, 8 incisions of one cm length were made on the dorsal skin of rats, and subcutaneous pockets were created with blunt, curved scissors. Sterile GelMAG or GDP hydrogels were prepared in a cylindrical PDMS mold (5 mm diameter, 2 mm depth), lyophilized, and UV‐treated. The hydrogels were implanted into the pockets before the incisions were closed with 4‐0 polypropylene sutures (Ad Surgical). On days 7 and 28 after implantation, the rats were euthanized by CO_2_ asphyxiation, and hydrogels were explanted with surrounding tissue for immunohistological analysis or biodegradation measurements.

To assess in vivo hemostatic efficacy using a liver hemorrhage model, rats received the same general anesthesia and underwent a median laparotomy, which exposed the liver and surrounding area. A 2 mm deep puncture was performed on the liver and immediately treated with sterile GelMAG or GDP prepolymer solutions that were photocured into a hydrogel. Sterile prepolymer solutions were prepared with methods used for in vitro biocompatibility, and the solution was stored at 37 °C until surgery. After the injury was prepared, the sterile precursor was applied with full wound coverage and photocrosslinked to form a hydrogel. An injury group with no hydrogel treatment was used as a control. Filter paper was used to collect blood for 10 min after injury and was later dried to measure total blood loss. Afterward, the abdominal wound was closed by suturing (4‐0 polypropylene), first the peritoneum and then the abdominal skin. After 14 days, the rats were euthanized, and hydrogels with surrounding tissue were explanted for immunohistological analysis.

In vivo hemostasis was also evaluated on a rat tail amputation model, where the rats received general anesthesia before 6 cm of the tail was cut. GelMAG or GDP prepolymer solution was immediately applied and photocured to form a hydrogel over the injury, while the control group received no hydrogel treatment. Blood loss was collected on filter paper for 10 min. Rats were euthanized before recovering from anesthesia.

Immunohistology was performed on hydrogel‐tissue interfaces to assess biocompatibility through the natural inflammatory response. Explanted hydrogels were first fixed in 4% (v/v) paraformaldehyde for 4 h and then left in 15% followed by 30% (w/v) sucrose solution at 4 °C overnight. Hydrogels were embedded in Optimal Cutting Temperature (O.C.T) compound, frozen in liquid nitrogen, and then sectioned with 10 µm thickness using a Leica CM1950 cryostat. The sections were contained on positively charged slides for H&E and immunostaining. Immunostaining was performed using anti‐CD3, anti‐CD45, anti‐CD68 as primary antibodies (Abcam) with Alexa Fluor 594‐conjugated Goat anti‐Rabbit IgG (H+L) (Invitrogen) as a secondary antibody. All samples were stained with DAPI, and fluorescence imaging was performed using an AxioObserver Z1 inverted microscope (Zeiss).

### In Vivo Efficacy of GDP Hemostatic Sealant Using the Porcine Lung Laceration Model

The pig lung laceration model was conducted at the University of California, San Diego (UCSD) under protocol S22108 using previously described methods.^[^ ^17^ ^]^ A right thoracotomy was conducted at the fourth intercostal space using a muscle‐sparing technique after preparing a 4–6 cm chest incision. Once the right top lobe of the pig lung was accessed, a laceration of 15 mm length and ≈3 mm depth was created using a surgical scalpel. We observed excessive air leaks and exsanguination at the incision site. The animals were placed on short periods of apnea (15–30 s) while GDP sealant (prepared with 20% w/v GelMAG, 0.1% w/v DMA, and 2% v/v pDDA) or Tisseel was applied to the injury. Air‐tight sealing was confirmed by submerging the thoracic cavity in saline and monitoring for air bubbles after increasing ventilation pressure in the lungs to 15–25 cmH_2_O. Once the injury was sealed, the intercostal space and the skin were separately closed using polypropylene sutures (3–0). The skin incision was set one intercostal space (i.e., fifth) caudal from the thoracotomy in order to prevent a postoperative pneumothorax, and the lungs were gently inflated during final closure of the thorax. The animals were extubated, and the thoracostomy tube was removed post‐operatively. Then, the skin wound was completely closed with an absorbable monofilament subcuticular suture. Once the injury was covered with a Tegaderm bandage, a thoracic ultrasound was conducted, and a linear surface ultrasound probe was used to evaluate for any radiographic signs of pneumothorax (through the occurrence of pleural line, A‐lines, B‐lines, etc.).

After 14 days post‐operation, the animals were sacrificed for testing the sealing efficacy and for IHC analysis. Prior to animal sacrifice, the tidal volume was increased in the intubated pigs, and ultrasound was conducted to measure hydrogel detachment from the injury through signs of pneumothorax. After animal sacrifice, the chest cavity was opened to expose the sealed laceration site, and tidal volume was again increased to monitor hydrogel detachment using PASCO Capstone software to record pressure in the lungs. Afterward, the GDP sealant and Tisseel hydrogels were explanted with surrounding lung tissue and processed for IHC analysis.

### Statistical Analysis

Statistical analysis was conducted on all numerical data using GraphPad Prism (version 8.4.3). Results were presented as mean ± standard deviation (S.D.). For two groups of data, an unpaired Student's *t*‐test was performed. For more than two groups of data, an analysis of variance (ANOVA) test was performed, followed by a Tukey's honestly significant difference (HSD) test for multiple comparisons within the groups. A one‐way ANOVA test was performed when there was one independent variable and one dependent variable, whereas a two‐way ANOVA test was performed when there were two independent variables and one dependent variable. The differences between groups were considered to be significant at ^*^ *p* < 0.05, ^**^ *p* < 0.01, ^***^ *p* < 0.001, ^****^ *p* < 0.0001. All individual‐level data are available in Data file S1 (Supporting Information).

## Conflict of Interest

N.A. is a co‐founder of, holds equity in, and has served as a consultant for GelMEDIX Inc. The remaining authors declare no conflict of interest.

## Author Contributions

S.J., A.B., and N.A. conceived the project and experimental design. S.J. conducted characterization, mechanical, adhesion, antibacterial, hemostatic, and biocompatibility testing. S.J., T.I., and N.K. designed and conducted the rat surgeries. S.J., J.A.B., and G.Z.C. designed and conducted the pig surgeries. S.J. prepared the schematics and manuscript, which was reviewed and revised by all authors. N.A. supervised the project.

## Supporting information



Supporting Information

Supplemental Movie 1

Supplemental Movie 2

Supporting Information

## Data Availability

The data that support the findings of this study are available in the supplementary material of this article.
