# Supplementary material for: Molecular Design of a Naturally Derived Hemostatic Sealant with Prolonged Antimicrobial Activity for Repairing Elastic Organ Injuries
Source: Adv Sci (Weinh). 2025 Sep 14;12(40):e06466. doi: 10.1002/advs.202506466 (PMC12561306; doi:10.1002/advs.202506466)
Supplement: Supplementary file 1 — Supporting Information [file ADVS-12-e06466-s004.docx]

**Supplementary Information for:**

**Molecular design of a naturally derived hemostatic sealant with prolonged antimicrobial activity for repairing elastic organ injuries**

Saumya Jain^1^, Avijit Baidya^1^, Joshua A. Boys^2^, George Z. Cheng^3^, Taichiro Imahori^4^, Naoki Kaneko^4^, Nasim Annabi^1,5*^

^1^ Department of Chemical and Biomolecular Engineering, University of California, Los Angeles, Los Angeles, California 90095, United States

^2^ Division of Cardiothoracic Surgery, Department of Surgery, University of California, San Diego, La Jolla, CA 92093, United States

^3^ Division of Pulmonary, Critical Care and Sleep Medicine, Department of Medicine, University of California, San Diego, La Jolla, CA 92093, United States

^4^ Division of Radiological Sciences, David Geffen School of Medicine, University of California, Los Angeles, Los Angeles, California 90095, United States

^5^ Department of Bioengineering, University of California, Los Angeles, Los Angeles, California 90095, United States

* Correspondence should be addressed to:

Dr. Nasim Annabi, PhD, E-mail: [nannabi@ucla.edu](mailto:nannabi@ucla.edu)

**Supplementary Materials include:**

**Figure S1.** ^1^H NMR of gelatin and GelMAG.

**Figure S2.** ^1^H NMR of dopamine and DMA.

**Figure S3.** ^1^H NMR of all compositions of prepolymers and hydrogels.

**Figure S4.** FTIR spectra of GDP hydrogels.

**Figure S5.** Ultimate tensile strength of GDP hydrogels.

**Figure S6.** Energy loss of GDP hydrogels after cyclic compression.

**Figure S7.** *In vitro* wound closure adhesion energy of GDP hydrogels.

**Figure S8.** Bacterial concentration and log reduction determined using spread plate method.

**Figure S9.** MIC of GDP and ciprofloxacin.

**Figure S10.** Representative images of live/dead stained bacteria after treatment.

**Figure S11.** Zeta potential measurements depicting pDDA release from GDP hydrogel.

**Figure S12.** Representative live/dead and actin/DAPI images after 1 day of incubation with GDP.

**Figure S13.** Cell number after incubation with GelMAG or GDP.

**Figure S14.** Representative images of *in vitro* hemostatic test with GDP hydrogels.

**Figure S15.** Absorbance values of hemoglobin during *in vitro* hemostatic test.

**Figure S16.** Swelling ratio of GDP hydrogels.

**Figure S17.** H&E images of GelMAG hydrogel and tissue on days 7 and 28 post-implantation.

**Figure S18.** Immunostaining of GelMAG-tissue interface on days 7 and 28 post-implantation.

**Figure S19.** Body weight and freedom from pneumothorax of pigs after lung laceration procedure.

**Other Supplementary Materials for this manuscript:**

## Movie S1. *Ex vivo* burst pressure testing of GDP on 20 mm laceration.

## Movie S2. *Ex vivo* burst pressure testing of GDP on 20 mm puncture.

##

**Figure S1. ^1^H NMR spectrum of gelatin and GelMAG prepolymer.** Magnified portion of the spectrum indicated emergence of methacryloyl proton peaks on GelMAG after methacrylation of gelatin.

**Figure S2.** **^1^H NMR spectrum of dopamine and DMA.** Magnified portion of the spectrum indicated emergence of methacryloyl proton peaks on DMA after methacrylation of dopamine.

**Figure S3. ^1^H NMR of the prepolymer and hydrogels of GelMAG, GP, GD, or GDP.** Magnified portion of the spectrum indicated the methacryloyl proton peaks on prepolymers and hydrogels.

**Figure S4. FTIR spectra of G, GP, GD, and GDP hydrogels.** The spectra depicted potential chemical interactions within the matrix, including hydrogen bonding and cation-π bonding.

**Figure S5. Ultimate tensile strength of GDP hydrogels prepared with varying amounts of pDDA and DMA.** Data are represented as mean ± SD. Analysis by two-way ANOVA with Tukey’s post-hoc multiple comparisons test. *P < 0.05, **P < 0.01, ***P < 0.001. n=3 per biological group.

**Figure S6. Energy loss after 12 rounds of cyclic compression on GDP hydrogels prepared with varying amounts of pDDA and DMA.** Data are represented as mean ± SD. Analysis by two-way ANOVA with Tukey’s post-hoc multiple comparisons test. *P < 0.05. n=3 per biological group.

**Figure S7. *In vitro* wound closure adhesion energy of GDP hydrogels prepared with varying amounts of pDDA and DMA.** Data are represented as mean ± SD. Analysis by two-way ANOVA with Tukey’s post-hoc multiple comparisons test. *P < 0.05. n=3 per biological group.

**Figure S8. In vitro antibacterial evaluation of the hydrogels using a spread plate method.** (**A**) Representative images of agar plates used to calculate CFU after P. aeruginosa or MRSA were treated with either GDP hydrogels formed with varying concentrations of DMA and pDDA, broad-spectrum antibiotic ciprofloxacin, or commercial wound dressing AquaDerm^TM^. (**B**) Concentration of P. aeruginosa or MRSA after treatment calculated as CFU/mL based on a spread plate method. (**C**) Log reduction of bacterial viability after treatment relative to untreated control. Data are represented as mean ± SD. Analysis by one-way ANOVA with Tukey’s post-hoc multiple comparisons test. *P < 0.05, **P < 0.01, ***P < 0.001, ****P < 0.0001. n=3 per biological group. Scale bar: 1 cm.

**Figure S9. Minimum inhibitory concentration (MIC) of broad-spectrum antibiotic ciprofloxacin and GDP against either P. aeruginosa or MRSA after 24 h incubation.** Data are represented as mean ± SD. Analysis by one-way ANOVA with Tukey’s post-hoc multiple comparisons test. ***P < 0.001. n=3 per biological group.

**Figure S10. Representative live/dead stained images of either *P. aeruginosa* or MRSA after 5 days of either no treatment (control) or treatment with GelMAG, GP, GD, GDP, ciprofloxacin, or AquaDerm^TM^.** Live cells were stained green and dead cells were stained red. Scale bar: 100 µm.

**Figure S11. Zeta potential measurements of DI water that was incubated with either GelMAG or GDP hydrogels to monitor the rate of pDDA release from the hydrogel matrix.** Data are represented as mean ± SD. Analysis by one-way ANOVA with Tukey’s post-hoc multiple comparisons test. *P < 0.05, **P < 0.01, ****P < 0.0001. n=3 per biological group.

**Figure S12.** **Representative** **live/dead and actin/DAPI stained images of NIH3T3 cells after one day of incubation with GelMAG or GDP sealants.** Control represents cells without hydrogel exposure. Scale bar: 100 µm.

**Figure S13.** **Number of NIH3T3 cells per unit area after incubation with GelMAG or GDP after 1 and 5 days.** Control represents cells without hydrogel exposure. Data are represented as mean ± SD. Analysis by two-way ANOVA with Tukey’s post-hoc multiple comparisons test. n=3 per biological group.

**Figure S14. Representative images for the *in vitro* hemostatic test performed on all GDP compositions with various concentrations of pDDA and DMA**, **using fresh whole blood to measure clotting time.** The images for the following conditions are also shown in Fig. 4A: Whole blood, GelMAG, GelMAG with DMA (GD), GelMAG with 2% pDDA (GP), and GelMAG with DMA and 2% pDDA (GDP).

**Figure S15. Absorbance values of hemoglobin during *in vitro* hemostatic test on fresh whole blood exposed to GDP hydrogels with various concentrations of pDDA and DMA.** The absorbance values for the following conditions are also shown in Fig. 4C: Whole blood, GelMAG, GelMAG with DMA (GD), GelMAG with 2% pDDA (GP), and GelMAG with DMA and 2% pDDA (GDP). Data are represented as mean ± SD. n=3 per biological group.

**Figure S16. Swelling ratio of GDP hydrogels containing various concentrations of pDDA and DMA**  **after incubation in DPBS at 37˚C.** Data are represented as mean ± SD. n=3 per technical group.

**Figure S17. H&E staining of the interface of GelMAG hydrogel and tissue.** Samples were explanted on days 7 and 28 after subcutaneous implantation. Scale bar: 100 µm.

**Figure S18. Representative immunostaining of GelMAG-tissue interface.** Samples were stained for hematopoietic cells (CD45, green), macrophages (CD68, red), and cell nuclei (DAPI, blue) on days 7 and 28 after subcutaneous implantation. Scale bar: 100 µm.

**Figure S19.** **Physical health of pigs with lacerate lungs that were treated with GDP hydrogel.** The pigs with GDP-sealed lung injuries were monitored to assess their physical health after the procedure by measuring (**A**) body weight after surgery (day 0) and during recovery (days 7 and 14 post-operation) and (**B**) freedom from pneumothorax assessed through thoracic ultrasound after surgery (day 0) and during recovery (day 14 post-operation). Data are represented as mean ± SD. n=3 per biological group.
